# Whole Brain Mapping of Neurons Innervating Extraorbital Lacrimal Glands in Mice and Rats of Both Genders

**DOI:** 10.3389/fncir.2021.768125

**Published:** 2021-10-29

**Authors:** Ying Zhai, Min Li, Zhu Gui, Yeli Wang, Ting Hu, Yue Liu, Fuqiang Xu

**Affiliations:** ^1^State Key Laboratory of Magnetic Resonance and Atomic and Molecular Physics, Key Laboratory of Magnetic Resonance in Biological Systems, Wuhan Center for Magnetic Resonance, Innovation Academy for Precision Measurement Science and Technology, Chinese Academy of Sciences, Wuhan, China; ^2^Centre for Brain Research, Department of Anatomy and Medical Imaging, Faculty of Medical and Health Sciences, University of Auckland, Auckland, New Zealand; ^3^Basic Medical Laboratory, General Hospital of Central Theater Command, Wuhan, China; ^4^Hubei Key Laboratory of Central Nervous System Tumor and Intervention, Wuhan, China; ^5^College of Life Sciences, Wuhan University, Wuhan, China; ^6^Shenzhen Key Laboratory of Viral Vectors for Biomedicine, The Brain Cognition and Brain Disease Institute (BCBDI), Shenzhen Institute of Advanced Technology, Chinese Academy of Sciences, Shenzhen, China; ^7^Shenzhen-Hong Kong Institute of Brain Science-Shenzhen Fundamental Research Institutions, NMPA Key Laboratory for Research and Evaluation of Viral Vector Technology in Cell and Gene Therapy Medicinal Products, Key Laboratory of Quality Control Technology for Virus-Based Therapeutics, Guangdong Provincial Medical Products Administration, Shenzhen, China; ^8^Center for Excellence in Brain Science and Intelligence Technology, Chinese Academy of Sciences, Shanghai, China; ^9^University of Chinese Academy of Sciences, Beijing, China; ^10^Wuhan National Laboratory for Optoelectronics, Huazhong University of Science and Technology, Wuhan, China

**Keywords:** extraorbital lacrimal gland, anatomy, neural tracing, neural circuits, CIL-LC-MS

## Abstract

The extraorbital lacrimal glands (ELGs) secret tears to maintain a homeostatic environment for ocular surfaces, and pheromones to mediate social interactions. Although its distinct gender-related differences in mice and rats have been identified, its comprehensive histology together with whole-brain neuronal network remain largely unknown. The primary objective of the present study was to investigate whether sex-specific differences take place in histological and physiological perspectives. Morphological and histological data were obtained *via* magnetic resonance imaging (MRI), hematoxylin-eosin (HE) staining in mice and rats of both genders. The innervating network was visualized by a pseudorabies virus (PRV) mediated retrograde trans-multi-synaptic tracing system for adult C57BL6/J mice of both genders. In terms of ELGs' anatomy, mice and rats across genders both have 7 main lobes, with one exception observed in female rats which have only 5 lobes. Both female rats and mice generally have relatively smaller shape size, absolute weight, and cell size than males. Our viral tracing revealed a similar trend of innervating patterns antero-posteriorly, but significant gender differences were also observed in the hypothalamus (HY), olfactory areas (OLF), and striatum (STR). Brain regions including piriform area (Pir), post-piriform transition area (TR), central amygdalar nucleus (CEA), medial amygdalar nucleus (MEA), lateral hypothalamic area (LHA), parasubthalamic nucleus (PSTN), pontin reticular nucleus (caudal part) (PRNc), and parabrachial nucleus, (PB) were commonly labeled. In addition, chemical isotope labeling-assisted liquid chromatography-mass spectrometry (CIL-LC-MS) and nuclear magnetic resonance spectroscopy (NMR spectroscopy) were performed to reveal the fatty acids and metabolism of the ELGs, reflecting the relationship between pheromone secretion and brain network. Overall, our results revealed basic properties and the input neural networks for ELGs in both genders of mice, providing a structural basis to analyze the diverse functions of ELGs.

## Introduction

The extraorbital lacrimal glands (ELGs) are the tubulo-acinar exocrine glands located below the outer ear in the subcutaneous tissue (Šemanjski et al., 2021). ELGs are well-known for their role in the maintenance of the homeostatic microenvironment for ocular surfaces by tear secretion, with less attention given to the importance of social behavior regulation and emotional process (Hirayama et al., 2013). The majority of current research on the ELGs is around dry eye model development *via* ELGs excision, together with general histological visualization of the ELGs without specific emphasis on sexual dimorphism (Shinomiya et al., 2018; Šemanjski et al., 2021). Only several previous studies have identified the pheromonal role of the exocrine gland-secreting peptides (ESPs) in the tear fluid that are specifically secreted by mice ELGs, which modulate sexual communication between rodents (Cavaliere et al., 2014). Intraspecies communication *via* pheromones plays an important role in social and sexual behaviors, which are crucial for survival and reproduction in animals (Tirindelli et al., 2009). However, the innervation patterns of ELGs in the brain and the exclusively neural basis of the complex interplay between the brain and the ELGs have not been mapped in detail to date.

This paper concentrates on systematic quantification, undertaking a detailed analysis of the direct innervations to ELGs across the whole brain between genders using the pseudorabies virus (PRV) mediated retrograde trans-multi-synaptic tracing system. PRV531-GFP and PRV724-dsRed were injected into different lobes of ELGs to explore if there are any functional discrepancies among lobes by labeling different upstream brain regions accordingly. Our results reveal the complex innervation patterns in the whole brain range and quantitatively compare their corresponding proportions. We found that the innervating distributions are similar for both genders along the anteroposterior (AP) axis but diverse for different subregions across genders. Furthermore, due to the lack of basic research of the extra-orbital gland, fatty acids and the energy metabolism of the extra-orbital gland were also studied by chemical isotope labeling-assisted liquid chromatography-mass spectrometry (CIL-LC-MS) and nuclear magnetic resonance spectroscopy (NMR spectroscopy). These analyses confirmed that ELG is a sex-hormone-dependent gland and may influence social behavior by pheromone secretion in different genders. The results further elucidate the regulation of the brain network on pheromone and hormone secretion and the influence of neurotransmitters on metabolism. Our results advance the identification of specialized neural networks from ELGs and provide tools to stimulate, examine, and determine the molecular mechanisms that bridge external stimuli with pheromonal secretion and specific behavioral responses.

## Materials and Methods

### Animals

All mice and rats were group-housed in a temperature- and humidity- controlled environment on a 12/12-h light/dark cycle and with *ad libitum* access to chow and water. Experiments were conducted on 13 adult C57BL6/J mice and 17 Sprague Dawley (SD) rats purchased from Hunan SJA Laboratory Animal Company, mice (male = 8; female = 5) with ages ranging from 8 to 32 weeks (20–31 g body weight) and rats (male = 14, female = 3) aged between 6 to 30 weeks (250–550 g body weight). Six mice (male = 3; female = 3) were used for viral injection with the rest used for histological staining, magnetic resonance imaging (MRI), and NMR. All the experiments with viruses were performed in bio-safety level 2 (BSL-2) laboratory and animal facilities. All surgery and experimental procedures were performed in accordance with the guidelines of the Animal Care and Use Committees at the Innovation Academy for Precision Measurement Science and Technology, Chinese Academy of Sciences.

### Surgery and Injections

Mice were anesthetized with sodium pentobarbital (80 mg/kg, i.p.), placed on their side above a heated surgical table. A thin layer of erythromycin ophthalmic ointment (0.5% w/w, Cisen Pharmaceutical Co., Ltd, China) was applied around the eye to protect it from prolonged operating-lamp exposure. The facial surgical site was shaved and cleaned with isopropyl alcohol. Metzenbaum scissors were used to make a 1-cm careful cutaneous incision along an axis of eyelid to ear, which exposed one side of the ELG. Extra care was taken not to injure the surrounding nerves and blood vessels. The thin connective tissue capsuling the ELG was gently torn and removed from the upper surface under a stereomicroscope to maximize penetration and accuracy of the viral injection. Six mice were used for viral injection and tracing, four relatively separated ELG injection sites were determined according to the longitudinal and vertical axis of the mice. Pseudorabies virus (PRV; Bartha stain; purchased from Dr. Fan Jia at the Brain Research Center, Wuhan Institute of Physics and Mathematics, Chinese Academy of Sciences) was injected with a hand-pulled glass micropipette (10–15μm in tip diameter) to at a steady rate. Two distant lobes of ELG were chosen during each injection for 1μL of PRV531-GFP (3 × 10^9^ pfu/ml) and 1μL of PRV724-dsRed (5 × 10^9^ pfu/ml) respectively, through which the corresponding functional capacities of different lobes could be visualized *via* was distinct labeling. The glass micropipette was left in place for an additional 1 min before slow withdrawal from the ELG. The surgical incisions were then sutured using 5-0 silk thread (Shanghai Pudong Jinhuan Medical Product Co., Ltd, China). Six PRV-injected mice were placed on electric heating blankets until full recovery, after which they were allowed to survive for 4.5d for optimal tracing and labeling before being sacrificed. In total, seven mice and eight rats were used for histological staining, following the procedure described above. However, these subjects underwent a blunt dissection for double side ELGs removal instead of viral injection, after which they were sacrificed by cervical dislocation. The weight of the ELGs has also been recorded for potential quantitative analysis.

### Perfusion and Tissue Cryosections

4.5 d after PRV injection, mice were deeply anesthetized with sodium pentobarbital (100 mg/kg, i.p.), then perfused transcardially with 0.01 M phosphate buffered saline (PBS, pH = 7.4, 5 mins) followed by 4% paraformaldehyde (PFA, Sigma-Aldrich, USA, MSDS, #158127) in 0.01M PBS (5 mins). Brain tissue was carefully collected and postfixed overnight in 4% PFA-PBS at 4, dehydrated in 30% sucrose in PBS at 4 for 48–72 h, and after which the whole brains were sectioned into 40 μm-thick coronal slices using a freezing microtome (CryoStar NX520 cryostat, Thermo Scientific, San Jose, CA, USA). All continuous brain slices were sequenced and collected in a 24-well plate with cryoprotectant solution (50% PBS; 20% Glycerol; 30% Ethylene glycol) at −20 until further processing.

Mice and SD rats with ELGs removed for histology were fixed in 4% PFA-PBS at 4 °C for 24 h, dehydrated in 30% sucrose in PBS at 4 °C for 48–72 h, and after which whole ELGs were sectioned into 16 μm-thick coronal slices and mounted on gelatin-coated slides (Citotest Scientific Co., Ltd, China; #80312316161). All slides were stored at −20 °C until further processing.

### Double-Labeling Immunohistochemistry (IHC)

PRV-injected sliced brain sections were removed from cryoprotectant and rinsed in PBS three times thoroughly at room temperature (RT) on a rocker for free-floating double-labeling fluorescent IHC. Non-specific staining was blocked with PBS containing 10% normal goat serum (Wuhan Boster Biological Technology, Ltd., Wuhan, China, #AR1009), 2% Albumin bovine (BSA, BioFroxx, Germany, #4240G250G), and 0.4% Triton X-100 for 1h at RT on a rocker. The sections were then incubated with chicken anti-GFP (Abcam, USA, #ab13970; 1:1000) and rabbit anti-dsRed (Takara, USA, #632496; 1:1000) overnight at 4. The next day, sections were washed three times with PBS for 15 min and fluorophore-coupled with goat anti-chicken IgG Alexa 594 (Jackson ImmunoResearch, USA, #103-585-155; 1:500) and goat anti-rabbit (Jackson ImmunoResearch, USA, #111-545-003; 1:500) secondary antibodies for 2 h at 37. After 2 h, sections were washed twice with PBS containing 0.2% Triton X-100 (PBST) for 10 min and once with PBS. Finally, 2-(4-Amidinophenyl)-6-indolecarbamidine dihydrochloride (DAPI, Beyotime Biotech, Jiangsu, China, #C1002; 1:4000) was added to the stain cell nucleus for 10 min at RT, washed an additional three times with PBS, mounted with 70% glycerol on gelatin-coated glass slides (Citotest Scientific Co., Ltd, China; #80312316161), and sealed with nail varnish.

### Hematoxylin and Eosin Staining

All slides were washed twice with distilled water for 2 min before being immersed in Hematoxylin (Beyotime Biotech, Jiangsu, China, #C0105S-1) for 5–10 min, after which the slides were further washed with distilled water and then stained with Eosin (Beyotime Biotech, Jiangsu, China, #C0105S-2) for 30 s. Slides were rinsed for 30 s in distilled water and dehydrated in ascending alcohol solution (50, 70, 80, and 95% × 2, 100% × 2). All slides were then cover slipped with 70% glycerol and sealed with nail varnish. For quantification of ELG cell size, four glands were randomly selected, out of which three field images (magnification 40 ×) were randomly selected. Cells in each field were counted by two researchers in a blind manner. The average cell size was calculated based on the ratio of the cell number and field size.

### Magnetic Resonance Imaging (MRI) of Rats' ELGs

MRI experiments were conducted using the Bruker Biospec 70/20 USR small animal MR system (Bruker BioSpin MRI, Ettlingen, Germany) operating at 7.0 T. The rats were anesthetized with 1% pentobarbital sodium (80 mg/Kg, ip). A planar receive coil surface coil with a diameter of 20 mm was placed on top of the ELG (with the skin removed) and was utilized in combination with a detunable partial volume transmit coil (Bruker BioSpin MRI, Ettlingen, Germany). Rat ELG T2 anatomical reference scan in the horizontal plane was acquired using a spin echo (Turbo-RARE) sequence: Field of view (FOV) = 20 × 20 mm^2^; Matrix dimension (MD) = 256 × 256; Repetition time (TR) = 2,500 ms; Echo time (TE) = 33 ms; RARE factor = 8; Number of averages (NA) = 4; Spatial resolution = 0.07 × 0.0.07 × 0.5 mm^3^; and 8 slices without gaps.

### Imaging, Cell Counting, and Data Analysis

All slides were scanned using the virtual microscopy slide scanning system (VS120, Olympus, Japan). The divisions of brain regions and areas were mainly based on the Allen Mouse Brain Atlas (ARA2011). In general, the whole brain was divided into nine major regions, comprising the isocortex, olfactory areas (OLF), striatum (STR), pallidum (PAL), hypothalamus (HY), cortical subplate (CTXsp), midbrain (MB), pons, and medulla. Each major brain region was further subdivided into several discrete brain subregions for better distributional analysis. All related subregions and their abbreviations are listed in Supplementary Table 1.

For cell counting, the labeled presynaptic neurons (expressing either GFP and dsRed only) within each brain region or subregion were quantified respectively according to the ARA2011 in every brain section by the cell counter plugin in ImageJ. Meanwhile, their location details were classified and recorded in the reference atlas. The total number of the labeled neurons within the whole brain or a certain brain region were quantified by adding up the numbers of the GFP-labeled neurons (GFP+) or dsRed-labeled neurons (dsRed+) neurons within all involved brain areas. For precise quantitative comparison of the distribution patterns of the labeled neurons across genders, the normalization was performed relative to the total number of the labeled neurons in the whole brain/a certain brain region/a certain brain area, and the proportions of the whole-brain inputs/a certain brain region inputs/a certain brain area inputs were quantified and analyzed, respectively. Gender-specific differences were evaluated by the procedures described above, which were conducted in the same way for both male and female mice.

For statistical analyses, two-tailed unpaired Student's *t*-tests, one-way ANOVA tests followed by Bonferroni tests were used for determining statistical differences using Python (version 3.9), with the significance set at ^*^p < 0.05, ^**^p < 0.01 and ^***^p < 0.001. All data values were presented as mean ± SEM. The related statistics are listed in Supplementary Table 3.

### LC-MS

#### Chemicals and Reagents

All fatty acid standards were purchased from Sigma (St. Louis, MO, USA) and J&K Chemical (Beijing, China). Analytical grade formic acid (FA), ethyl acetate (EA), triethylamine (TEA), 2-chloro-1-methylpyridinium iodide (CMPI), and DMED were obtained from Sinopharm Chemical Reagent Co., Ltd. (Shanghai, China). The isotope labeling reagent of d4-DMED was synthesized in our lab with the procedures published previously (Hu et al., 2020). HPLC-grade acetonitrile (ACN) and methanol were purchased from TEDIA Co., Inc (Fairfield, OH, USA). Ultrapure water was purified by a Milli-Q apparatus (Millipore, Milford, CT, USA). Stock solutions of TEA (20 mmol/L), CMPI (20 mmol/L), DMED (40 mmol/L), and d4-DMED (40 mmol/L) were prepared in HPLC-grade ACN. Stock solutions of standard organic acids were prepared in HPLC-grade ACN with a concentration of 1.0 mg/mL for each. All stock solutions were stored at −20 °C until analyzed.

#### Metabolite Extraction

The ELG samples were harvested from Sprague Dawley male rats after they were euthanized. ELG samples were washed with 1 × phosphate-buffered saline (PBS) to free them of most of the blood and stored at −80 °C until further processing. Then, the frozen tissues were first weighed and ground in 1mL pre-cooled saline solution while keeping them in the ice-bath. After the addition of EA (2 mL), the mixture was vortexed for 3 min for the extraction of fatty acids. After extraction, the samples were centrifuged at 13,000 g for 15 min at 4 °C, and the supernatant was transferred to a new vial. The extraction procedure with EA was repeated twice, and the supernatants were combined and vortexed again. The transferred supernatants were dried under nitrogen gas and stored at −80 °C until further analysis.

#### Isotope Labeling Reaction

For labeling, the dried samples were dissolved in 200 μL ACN and then the sample was divided into two equal parts. Each part of the dissolved sample was added to 20 μL of TEA (20 mmol/L) and 10 μL CMPI (20 mmol/L). Then, the sample was mixed and incubated at 40 °C for 5 min. After that, 20 μL of DMED (40 mmol/L) or 20 μL d4-DMED (40 mmol/L) was added, and the solution was incubated for another 60 min at 40 °C. In the end, the resulting solution was dried under nitrogen and re-dissolved in 100 μL ACN/water (v/v, 1/9) and left for LC-Orbitrap MS analysis. To overcome the error caused by retention time drifts in LC analysis, retention indices were adopted for correction by adding straight-chain n-alkanoic acids (C5:0-C24:0) labeled by d4-DMED.

Labeled tissue extracts were analyzed using an LTQ-Orbitrap Elite mass spectrometer (Thermo Scientific, Waltham, MA, USA), coupled with a Dionex Ultimate 3000 UHPLC system (Thermo Scientific, Sunnyvale, CA, USA). An Acquity UPLC BEH C18 Column (2.1 × 50 mm, 1.7 m, Waters, Milford, CT, USA) was used for HPLC separation with a flow rate of 0.4 mL/min at 40 °C. FA in water (0.1%, v/v, solvent A), and ACN (solvent B) were used as mobile phases. The gradient elution profile was as follows: 0–5 min at 5% B, 5–48 min from 5 to 95% B, 48–53 min at 95% B, 53–55 min from 95 to 5% B, and 55–60 min at 5% B. The injection volume was 10 L. All mass spectra were collected in the positive ion full scan mode in the range of m/z 180–650 at a resolution of 60,000. ESI conditions were capillary temperature, 350 °C; spray voltage, 3.5 kV; sheath gas flow, 35 arbitrary; auxiliary gas flow, 15 arbitrary; heater temperature, 300 °C.

#### Data Processing and Analysis

The entire list of centroid peaks with collected information (e.g., retention times, m/z values, peak intensities) was exported from Thermo Xcalibur 2.1 Software (Thermo Fisher Scientific, Inc., Waltham, MA, USA). Retention time (RT) was calibrated by retention indices (RIs) as reported [28] to overcome the drifting of retention times. Peak pair extraction was operated with 4.025 Da mass difference, similar peak intensities, and RIs by using in-house MATLAB-based software. Prospective molecular formulas of DMED-labeled fatty acids were generated based on the accurate m/z using the Thermo Xcalibur 2.1 Software. Mass tolerance of 5.0 mDa was set, and the elements C, H, N, and O were used. Based on accurate mass and retention time, positive fatty acid identification was performed using an in-house chemically labeled standard library (http://59.110.238.58/search).

### Acquisition of NMR Spectra and Data Analysis

All NMR spectra were acquired at 298K by BrukerAvance III 500 MHz NMR spectrometer (BrukerBiospin, Germany). The POCE ([1H-13C]-NMR) pulse sequence was used to detect the enrichment of 13C in the ELGs extract. The following parameters were used in the acquisition process: echo time:8 ms; sweep width:20 ppm; repetition time:20 s; the number of scans:32; acquisition data-64 K. The collected data is processed by Topspin 2.1 (Bruker Biospin, GmbH, Rheinstetten, Germany) and a homemade software NMRSpec (Liu et al., 2017). After preprocessing the NMR spectra, the average spectra are obtained by MATLAB. [1-^13^C] Glucose Infusion and Tissue Extraction.

All animals were injected with [1-^13^C] glucose (Qingdao Tenglong Weibo Technology co., LTD, Qingdao, P.R. China) to detect the enrichment of metabolites in the ELGs. Rats were fasted 15–18h before the experiment to obtain a higher enrichment rate of ^13^C in the metabolites and to enhance detection sensitivity.

On the day of the experiment, A 24-gauge injection needle was connected to a PE50 catheter (Instech, PA, USA) and inserted into the tail vein of the rat, and then the infusion tube was connected to a swivel, and ^13^C-labeled glucose was infused through a pump (Fusion 100, Chemyx, TX, USA) for 2 min. After 20 min of free movement in the cage, the rats were deeply anesthetized with isoflurane, and all animals were euthanized with head-focused microwave irradiation. (1 KW, Tangshan Nanosource Microwave Thermal Instrument Manufacturing Co. Ltd., Heibei, PR China). The extraorbital glands on both sides of the rat's head were peeled off, weighed, and the metabolites were extracted. The extraction method followed that used in previous experiments (Wu et al., 2020; Guo et al., 2021), especially the use of chemical TMSP [3-(Trimethylsilyl) propionic-2,2,3,3-d4 acid sodium salt], which was used as the inner standard chemical, which was necessary for sample detection.

## Results

### Overview of the Neurons Innervating ELGs Across the Whole Brain

**Figure 1:**
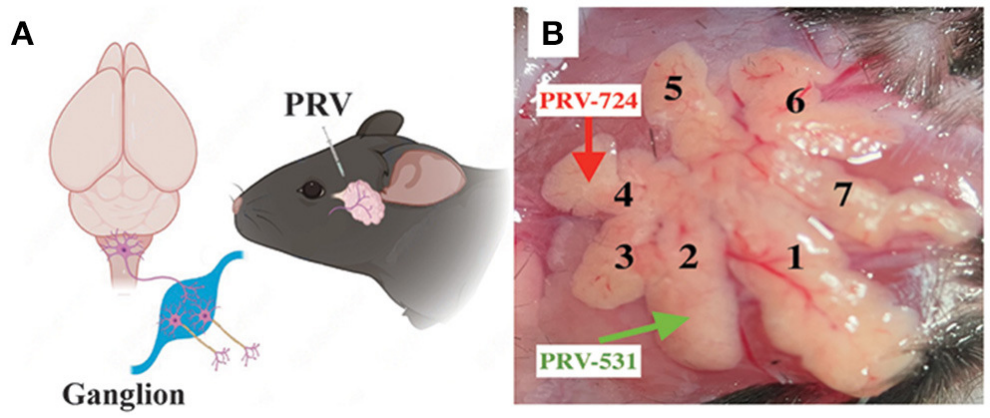
Experimental procedures for the cell-type-specific retrograde trans-multi-synaptic tracing of whole brain. **(A)** Simplified drawing of ELG's geographic location on mice, and the general principle of PRV tracing from peripheral ELGs to the central nervous system. **(B)** Experimental design showing the injection site for C57BL6/J (example injection sites combined lobe No. 2 and lobe No. 4, with PRV531-GFP and PRV724-dsRed injected respectively).

**Figure 2:**
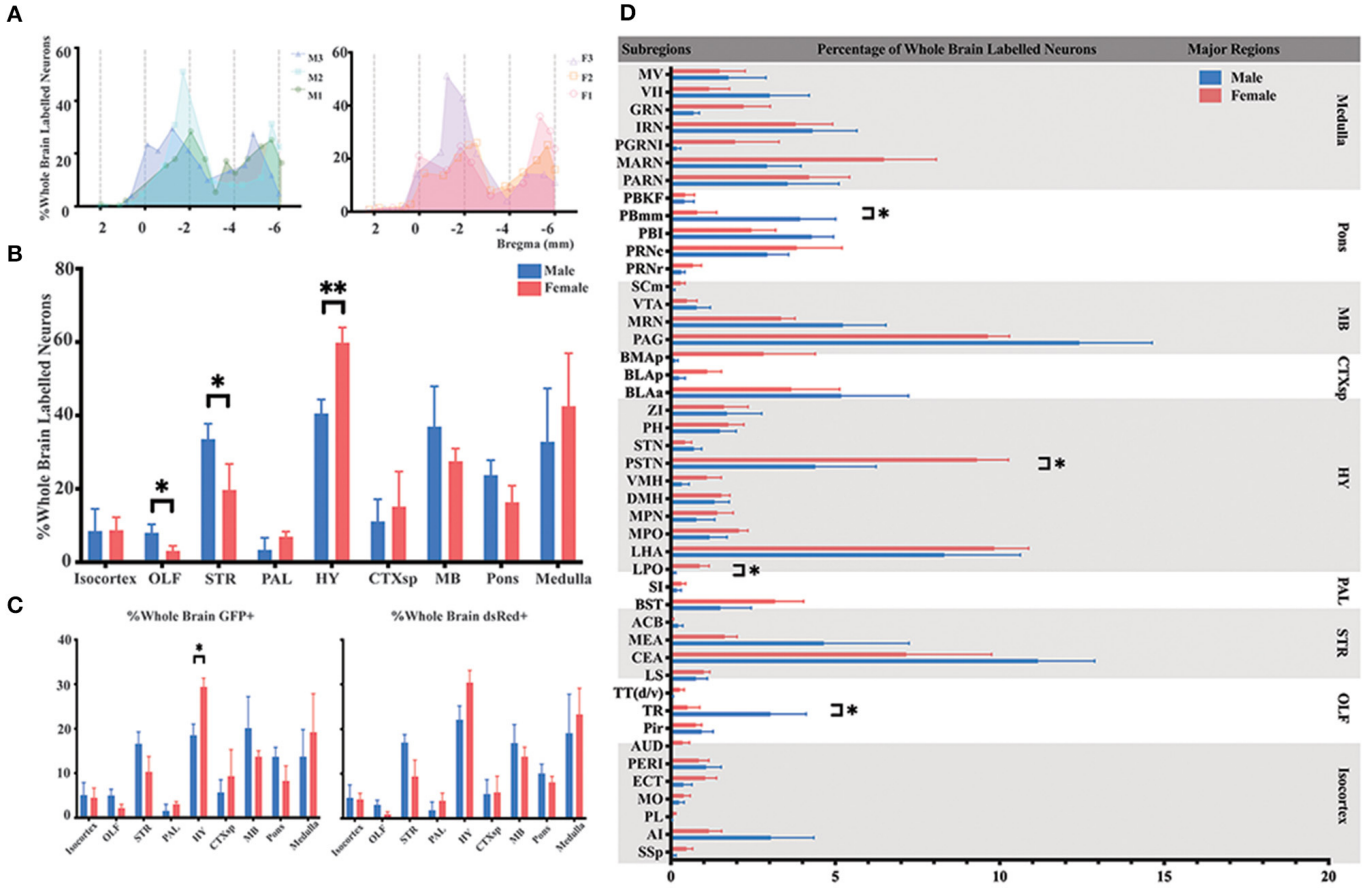
Distribution patterns of whole-brain inputs to the ELGs. **(A)** Whole-brain distribution of all labeled neurons along the AP axis. Colored lines, input distribution for the individual mouse; colored line with a shaded area under it, average labeled distribution (left, male; right, female). M1–3, male mouse 1–3; F1 – 3, female mouse 1–3. **(B)** Whole-brain distribution of the labeled neurons within nine major brain regions from bilateral hemispheres, quantified with all slices. **(C)** The proportion of GFP+ and dsRed+ in nine major brain regions from bilateral hemispheres, quantified with all slices (left, GFP+; right, dsRed+). **(D)** Whole-brain distribution of all labeled neurons within 45 subregions in bilateral hemispheres (only significant differences have been labeled on the corresponding top of the bars, otherwise n.s.). GFP+, dsRed+, n.s., no significant difference; *p < 0.05 and **p < 0.01.

To identify the whole-brain neurons innervating ELGs, C57BL6/J mice (male = 3; female = 3) and PRV-based retrograde trans-multi-synaptic tracing system were utilized with simplified experimental procedures along with PRV tracing principle shown in Figure 1. 4.5 days of PRV transmission was employed to best visualize the whole brain pattern of infection based on our initial experimental setup, which has 2.5 d PRV transmission with infection only reaching up to the level of brainstem and medulla oblongata even with longer IHC antibody incubation (see Supplementary Figure 1). For quantitative analysis, we counted 1,330–11,267 GFP-labeled neurons (GFP+) and 587–4,263 dsRed-labeled neurons (dsRed+) in each brain across genders, with male 3# and female 1# having relatively fewer overall counts than others (see Supplementary Table 2 for specific data values). Our results revealed that ELGs received extensive overall innervations (i.e., sum of GFP+ and dsRed+) from the brain regions along the AP axis (Figure 2A). To compare the overall labeling pattern across nine major brain regions in both genders, the number of labeled neurons within each brain region from bilateral hemispheres was normalized related to the total number of all labeled neurons in the whole brain. Our results revealed that both male and female subjects showed a similar trend of labeled pattern (Figures 2A,B). Overall, most of the labeled neurons in male mice were observed within the HY (20.30 ± 1.95%), followed by MB (18.51 ± 3.72%), STR (16.79 ± 1.44%), medulla (16.41 ± 4.90%), and pons (11.87 ± 4.90%). The remaining regions, such as CTXsp (5.56 ± 1.93%), isocortex (4.85 ± 1.79%), OLF (4.01 ± 0.89%), and PAL (1.68 ± 1.04%) were sparsely labeled (Figure 2B; see Supplementary Table 3 for full list of data values).

For females, the majority of the labeled neurons also arose from HY (29.93 ± 1.51%), followed by medulla (21.28 ± 4.74%), MB (13.78 ± 1.11%), STR (9.85 ± 2.26%), and pons (8.19 ± 1.60%). Along with minor contributions from CTXsp (7.58 ± 3.21%), isocortex (4.38 ± 1.14%), PAL (3.49 ± 0.84%), and OLF (1.54 ± 0.53%) (Figure 2B; see Supplementary Table 3 for full list of data values). To further elucidate the distribution patterns of GFP+ and dsRed+ within each major brain region across genders, the number of the GFP+ and dsRed+ within each brain region from bilateral hemispheres was normalized relative to the total number of the GFP+ and dsRed+ in the whole brain respectively. Our results showed that both males and females have an overall similar trend of GFP+ and dsRed+ distribution, except one significant difference was observed in the HY of GFP+ (18.52 ± 2.50% for males; 29.43 ± 1.93% for females, *P* < *0.05*) after statistical analysis (Figure 2C; see Supplementary Table 4 for a full list of data values). The overall labeling pattern was further explored in a total of 45 brain subregions in both genders (Figure 2D). Four subregions have displayed the significant difference of all labeled neurons between males and females, which include post-piriform transition area (TR, 3.03 ± 1.08% for males vs. females, 0.50 ± 0.37%, *P* < *0.05*), lateral preoptic area (LPO, 0.09 ± 0.05% for males vs. females, 0.87 ± 0.29%, *P* < *0.05)*, parasubthalamic nucleus (PSTN, 4.39 ± 1.84% for males vs. females, 9.31 ± 0.96%, *P* < *0.05*), and parabrachial nucleus, medial division, medial meidal part (PBmm, 3.93 ± 1.08% for males vs. females, 0.80 ± 0.59%, *P* < *0.05*) (Figure 2D; see Supplementary Table 3 for a full list of data values). The top three sources of labeled neurons in males originated from the periaqueductal gray (PAG), central amygdalar nucleus (CEA), and lateral hypothalamic area (LHA), all of which contributed over 32.24% of whole-brain labeled neurons to the ELGs in total. The top three in females were from LHA, PAG, and PSTN, and over 28.2% of whole-brain labeled neurons arose from these subregions in total (Figure 2D; see Supplementary Table 3 for a full list of data values).

### Innervations From Bilateral HY to the ELGs

**Figure 3:**
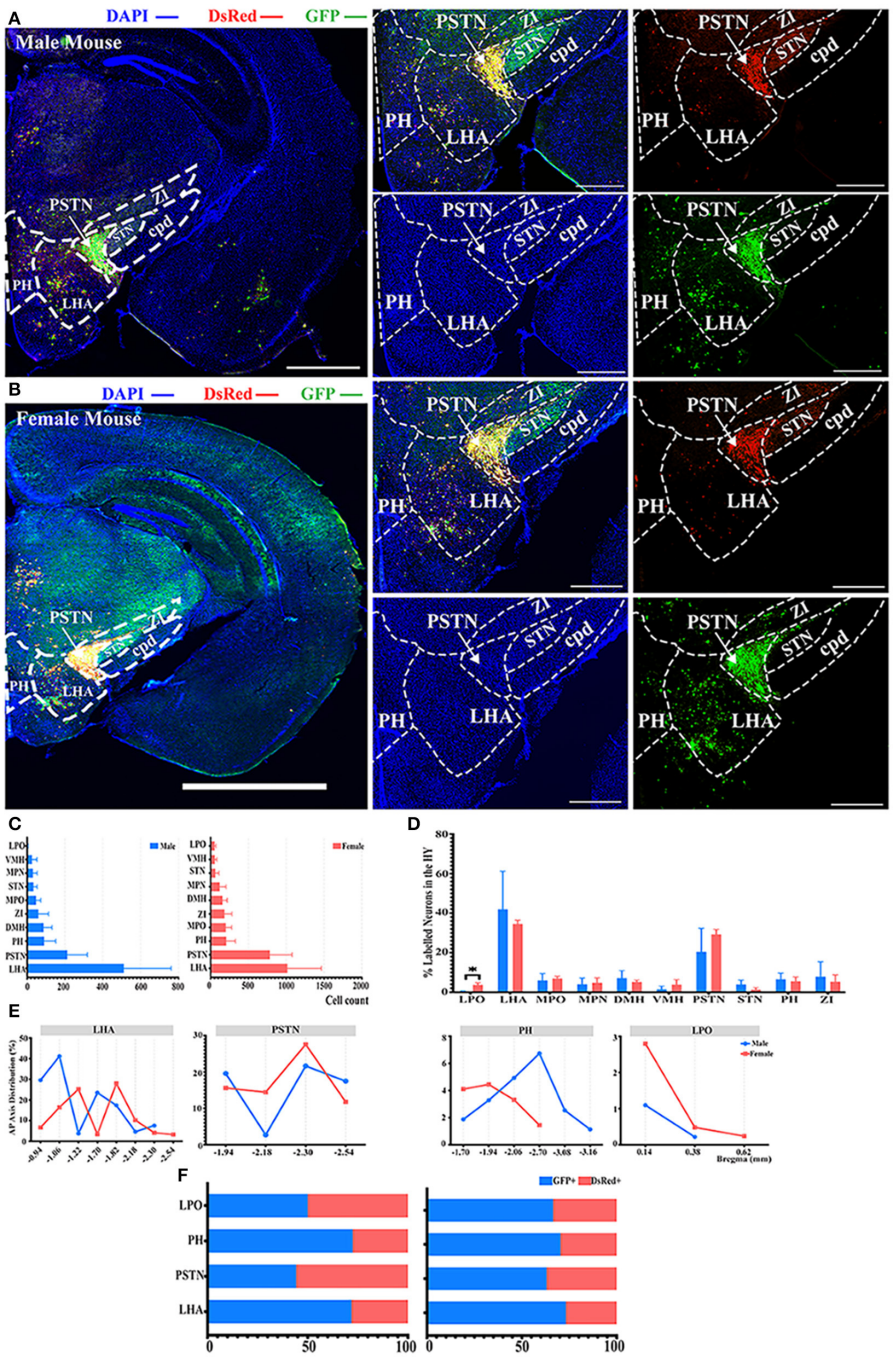
Distribution patterns of labeled neurons in HY. **(A,B)** Representative images showing labeled neurons from the mouse ipsilateral HY to the ELGs. Scale bar: 1 mm (left lower magnification images) and 200 μ*m* (for right higher magnification images); (**A**, male; **B**, female). **(C)** Numbers of the labeled neurons (GFP+ and dsRed+) in different subregions of HY (left, male; right, female). **(D)** Distribution of the labeled neurons within HY (significant difference labeled on the bar top, otherwise n.s.). **(E)** Distribution patterns of the labeled neurons along the AP axis in different subregions of HY. **(F)** Distribution of GFP+ and dsRed+ within subregions of HY (left, male; right, female). GFP+, PRV-GFP positive neurons; dsRed+, PRV-dsRed positive neurons., n.s., no significant difference; *p < 0.05.

Most of the innervations to the ELGs stemmed from the HY in both genders, which can be subdivided into the lateral preoptic area (LPO), LHA, medial preoptic area (MPO), medial preoptic nucleus (MPN), the dorsomedial nucleus of the hypothalamus (DMH), ventromedial nucleus of the hypothalamus (VMH), PSTN, subthalamic nucleus (STN), posterior hypothalamic nucleus (PH), and zona incerta (ZI) (Figures 2D, 3). Our results revealed that the overall labeled neurons in females outnumbered males by 2.6 to 1 (Figure 3C). Among these HY subregions, the LHA, PSTN, and PH were the top three sources of labeled neurons in both males and females (Figures 3A–C). The number of labeled neurons within each subregion was normalized related to the total number of all labeled neurons in the HY. Our results illustrate that over 70.34% of labeled neurons arose from LHA, PSTN, and ZI in males, whereas approximately 70.78% of labeled neurons came from LHA, PSTN, and MPO in females (Figure 3D). All subregions followed a similar trend of labeled neurons with only one documented subregional significant difference found in the LPO (0.41 ± 0.35% for males vs. females, 3.79 ± 1.01%, *P* < *0.05*) between genders (Figure 3D; see Supplementary Table 3 for a full list of data values). We further analyzed the distribution patterns along the AP axis and the contribution of GFP+/dsRed+ within LHA, PSTN, PH, and LPO. Our results indicated that the proportion of labeled neurons along the AP axis also followed similar distribution patterns in LHA and PSTN among males and females (Figure 3E). Relatively fewer labeled neurons were found located in PH compared to the previous two, and the number of labeled neurons within this area peak at bregma −1.94 mm for females and −2.70 mm for males respectively along the AP axis (Figure 3E). LPO has a descending trend along the AP axis, with the fewest neurons found around bregma 0.38 mm of both genders (Figure 3E). In both males and females, the labeled neurons were mostly found to be GFP+ except in PSTN, where male mice have on average only 44.25% of GFP+ contribution (Figure 3F).

### Innervations From Bilateral OLF to the ELGs

**Figure 4:**
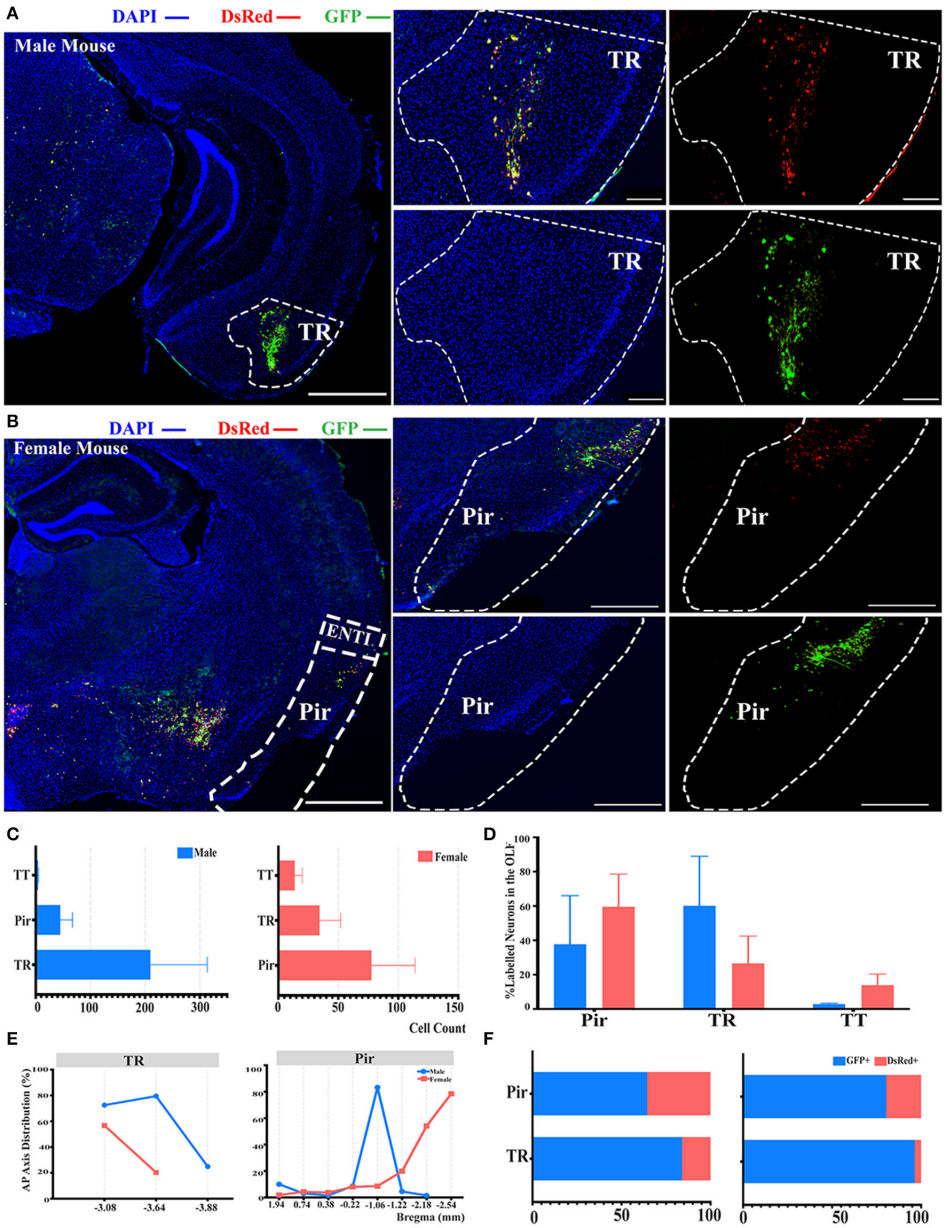
Distribution patterns of labeled neurons in OLF. **(A,B)** Representative images showing labeled neurons from the mouse ipsilateral OLF to the ELGs. Scale bar: 1 mm (left lower magnification images) and 200 μ*m* (right higher magnification image); (**A**, male; **B**, female). **(C)** Number of labeled neurons (GFP+ and dsRed+) in different subregions of OLF (left, male; right, female). **(D)** Distribution of the labeled neurons within OLF (significant difference labeled on the bar top, otherwise n.s.). **(E)** Distribution patterns of the labeled neurons along the AP axis in different subregions of OLF. **(F)** Distribution of GFP+ and dsRed+ within subregions of OLF (left, male; right, female). GFP+, PRV-GFP positive neurons; dsRed+, PRV-dsRed positive neurons. n.s., no significant difference.

Another significant difference of labeled neurons between genders was documented in the OLF when normalized and related to the total number of all labeled neurons in the whole brain, as indicated in Figure 2B. Hence, we conducted further investigation within the OLF by stratifying its labeled neuron intensity between different subregions. TR was the subregion with the most labeled neurons in males (60.10 ± 28.86%), while in females the majority of labeled neurons were found in Pir (59.57 ± 18.97%), although no statistical significance was observed (Figures 4A–D; see Supplementary Table 3 for the full list of data values). Total cell counts in all three subregions of the OLF in males outnumbered females by 2 to 1 (Figure 4C). Labeling patterns were similar between genders as no significant difference among these subregions has been found when normalized to the total number of all labeled neurons in the OLF (Figure 4D; see Supplementary Table 3 for the full list of data values). Seldom labeled neurons were found in taenia tecta (TT) (2.85 ± 0.48% for males vs. females, 13.89 ± 6.46%) of both genders, thus only the top two sources of labeled neurons from TR and piriform area (Pir) were analyzed for distribution patterns (Figure 4D; see Supplementary Table 3 for the full list of data values). A similar decreasing trend along the AP axis for the proportion of labeled neurons within TR between males and females was revealed (Figure 4E). In contract to Pir in males, most labeled neurons were found to be concentrated around bregma −0.22 mm to −1.22 mm, which accounted for over 83% of all labeled neurons in this region (Figure 4E). Females, by contrast, have an overall increasing trend of labeled neurons along the AP axis, with over 78% of all labeled neurons found around bregma −2.54 mm (Figure 4E). For the composition of GFP+ and dsRed+ within TR and Pir, the majority of the labeled neurons were GFP+, which contribute to 64 to 96% of all labeled neurons (Figure 4F).

### Innervations From Bilateral STR to the ELGs

**Figure 5:**
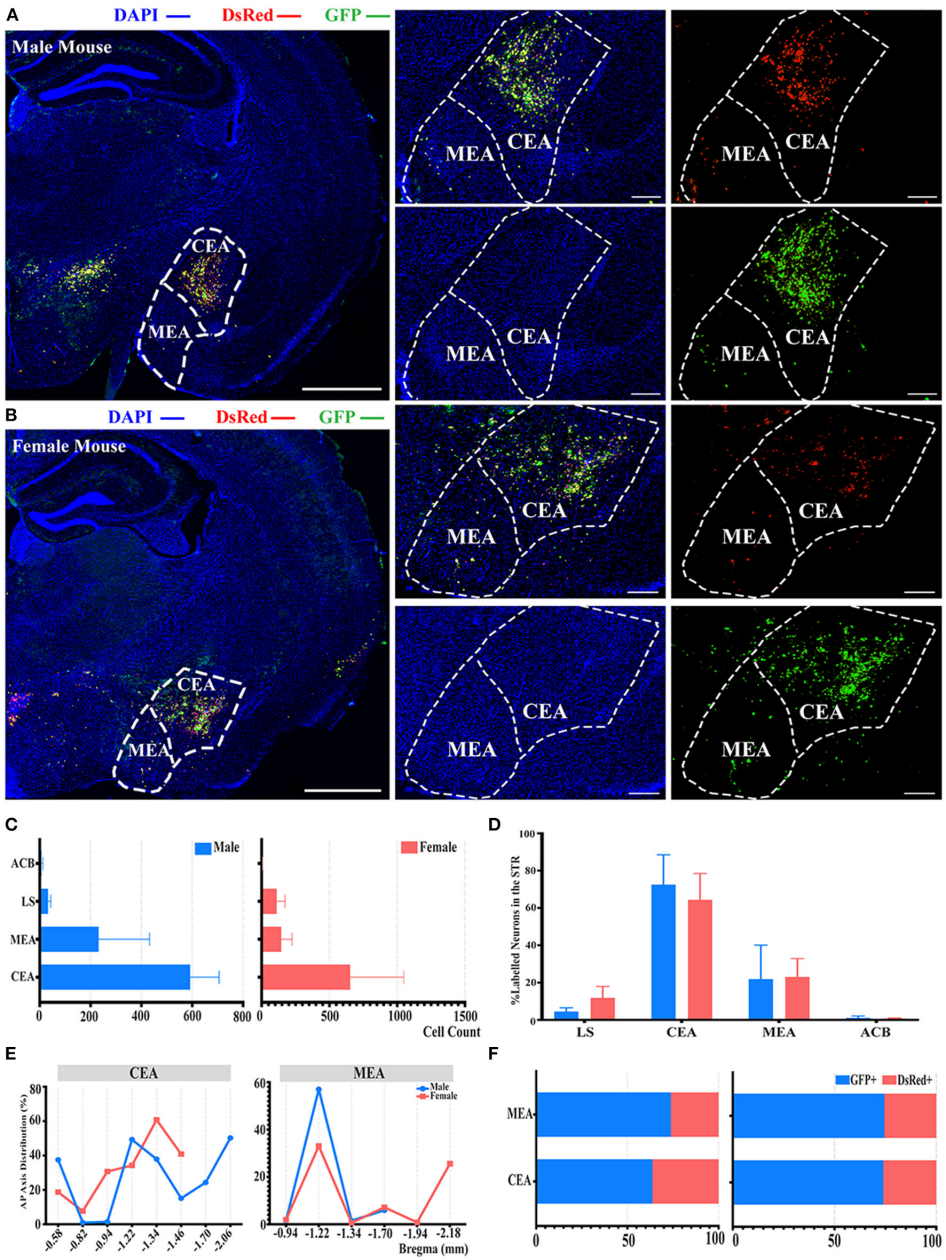
Distribution patterns of labeled neurons in the STR. **(A,B)** Representative images showing labeled neurons from mouse the ipsilateral STR to the ELGs. Scale bar: 1 mm (left lower magnification images), 200 μ*m* (right higher magnification image for male), and 500 μ*m* (right higher magnification image for female); (**A**, male; **B**, female). **(C)** Numbers of the labeled neurons (GFP+ and dsRed+) in different subregions of STR (left, male; right, female). **(D)** Distribution of the labeled neurons within STR (significant difference labeled on the bar top, otherwise n.s.). **(E)** Distribution patterns of the labeled neurons along the AP axis in different subregions of STR. **(F)** Distribution of GFP+ and dsRed+ within subregions of STR (left, male; right, female). GFP+, PRV-GFP positive neurons; dsRed+, PRV-dsRed positive neurons. n.s., no significant difference.

There was also another significant difference observed in the STR compared to the whole brain labeled neurons between males and females, as shown in Figure 2B. In the STR, the top two sources of labeled neurons were the CEA and medial amygdalar nucleus (MEA), which contributed to approximately 72 and 21% of STR labeled neurons in males, along with approximately 64 and 23% in females, respectively (Figures 5A–D). However, no statistically significant difference was observed in the normalization related to all labeled neurons in the STR of both genders (Figure 5D; see Supplementary Table 3 for the full list of data values). Less than 1% of labeled neurons were found to be located within nucleus accumbens (ACB) in both males and females (Figure 5D). For the distribution along the AP axis, both genders demonstrated similar labeling patterns by having a peak density of labeled neurons around bregma −1.22 to −1.34 mm for both CEA and MEA (Figure 5E). Together with previous results of GFP+/dsRed+ distribution, CEA and MEA also had a higher proportion of GFP+, ranging from 63 to 74% of the total number of labeled neurons in corresponding subregions (Figure 5F).

### Innervations From Bilateral Pons to the ELGs

**Figure 6:**
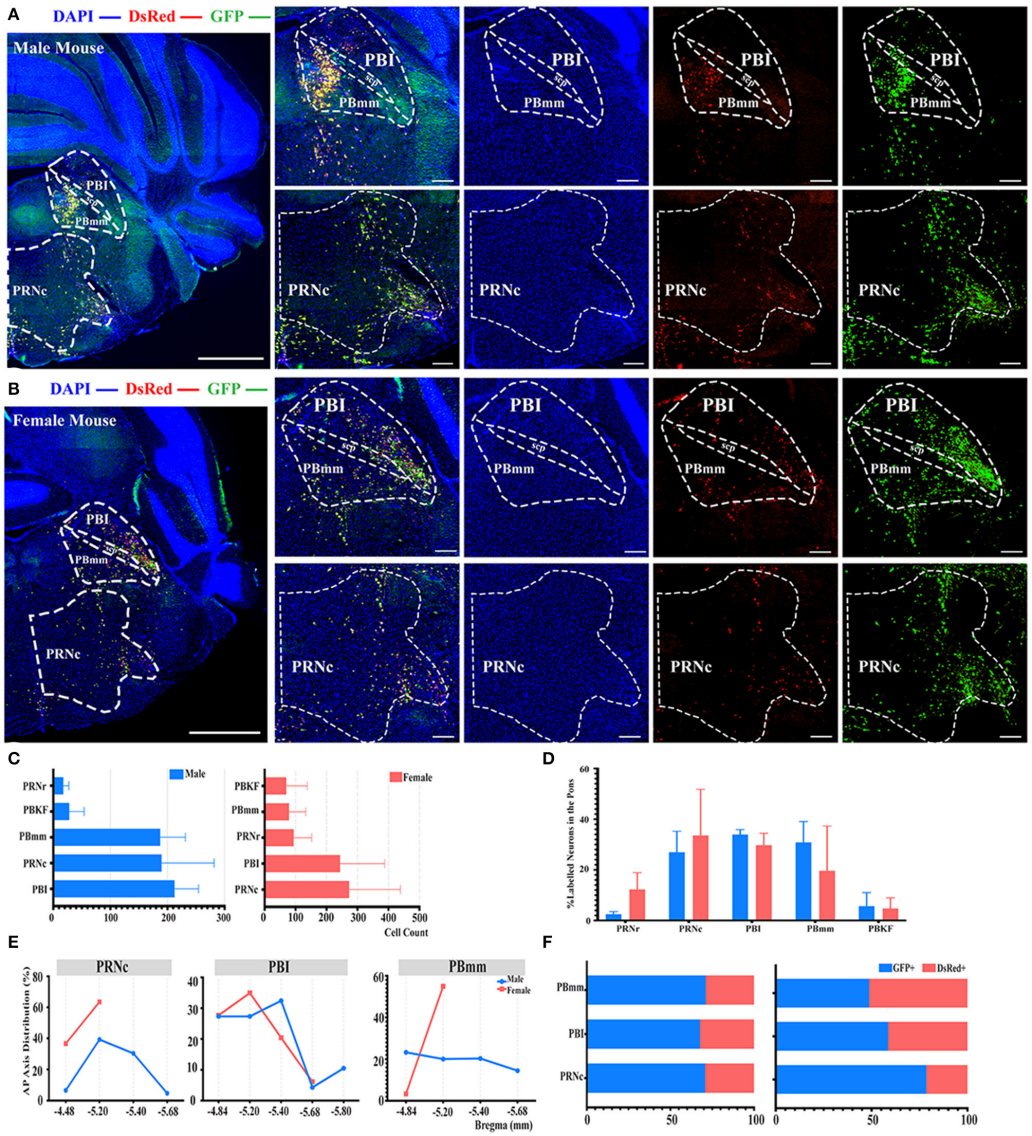
Distribution patterns of labeled neurons in the Pons. **(A,B)** Representative images showing labeled neurons from the mouse ipsilateral pons to the ELGs. Scale bar: 1 mm (left lower magnification images) and 200 μ*m* (right higher magnification images); (**A**, male; **B**, female). **(C)** Number of labeled neurons (GFP+ and dsRed+) in different subregions of Pons (left, male; right, female). **(D)** Distribution of the labeled neurons within Pons (significant difference labeled on the bar top, otherwise n.s.). **(E)** Distribution patterns of the labeled neurons along the AP axis in different subregions of Pons. **(F)** Distribution of GFP+ and dsRed+ within subregions of Pons (left, male; right, female). GFP+, PRV-GFP positive neurons; dsRed+, PRV-dsRed positive neurons. n.s., no significant difference.

Even though no significant difference was reported in the pons when normalized with whole brain labeled neurons, as shown in Figure 2B, a subregional significant difference was discovered in the PBmm between genders, with males having a substantially higher density of labeled neurons than females (3.93 ± 1.08% for males vs. females, 0.80 ± 0.59%, *P* < *0.05*; Figure 2D; see Supplementary Table 3 for the full list of data values). The results of our additional analysis within the pons displayed that the caudal part of the pontine reticular nucleus (PRNc) and the lateral division of the parabrachial nucleus (PBI) were the top two sources of labeled neurons in both genders (Figures 6A–C). Over 34, 30, and 27% of the total labeled neurons in males were derived from PBI, PRNc, and PBmm respectively (Figure 6D). Approximately 33, 30, and 19% of total labeled neurons in females came from PRNc, PBI, and PBmm respectively (Figure 6D). Similar to previous statistical analysis results, males and females had a similar tendency of labeling, as no significant difference was identified within these subregions when normalized and related to the total number of labeled neurons in the pons (Figure 6D). In terms of AP distribution in PRNc, both genders had a peak intensity of labeled neurons of around bregma −5.2 mm, and males showed a descending trend after the peak that was absent in female subjects (Figure 6E). For labeling patterns in PBI along the AP axis, a similar trend was noticed between genders with the most labeled neurons around bregma −5.2 mm (Figure 6E). However, PBmm exhibited a divergent trend of labeling patterns between genders, with males showing evenly distributed labeled neurons anterior-posteriorly, and females exhibiting sharply escalating labeled neurons from bregma −4.84 to −5.2 mm (Figure 6E). In males' PBmm, PBI, and PRNc, GFP+ neurons comprised approximately 71, 67, and 70% of the total neurons, respectively (Figure 6F). Females had 48, 58, and 78% of GFP+ in the PBmm, PBI, and PRNc, respectively (Figure 6F).

### Anatomy and Histology of the ELGs

**Figure 7:**
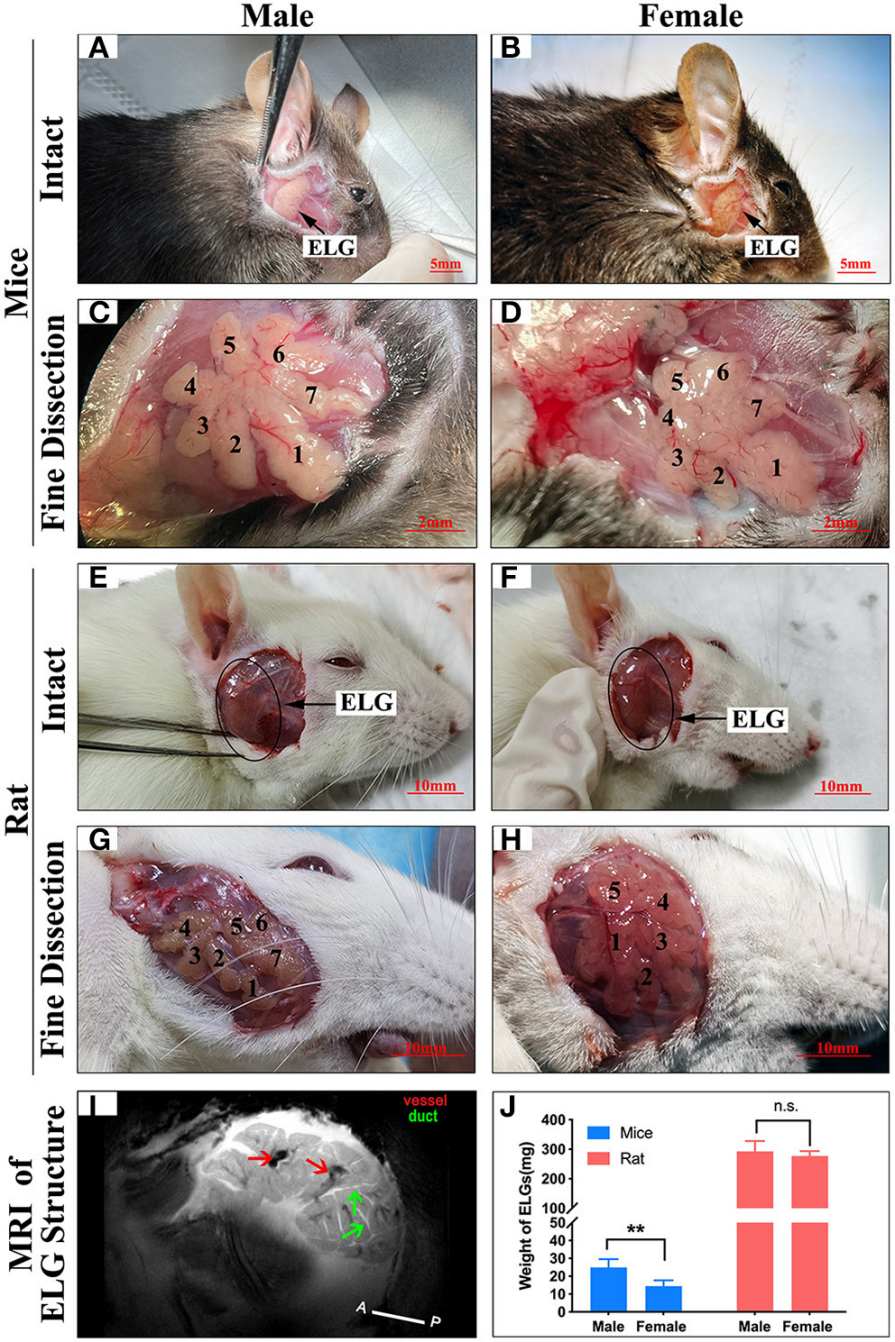
Anatomy of ELGs. Entire appearance on ELGs on the face of **(A)** Male mice (scale bar = 5 mm), **(B)** Female mice (scale bar = 5 mm), **(E)** Male rat (scale bar = 10 mm), **(F)** Female rat (scale bar = 10 mm). ELGs locations are labeled by an arrow and blank circle in mice and rats, respectively. Fine dissection of ELG on the face of **(C)** Male mice (scale bar = 2 mm), **(D)** Female mice (scale bar = 2 mm), **(G)** Male rat (scale bar = 10 mm), **(H)** Female rat (scale bar = 10 mm). (Figure is labeled on each lobe of ELG). **(I)** T2 anatomical MRI of rat's ELG (horizontal section). The red arrows point to the blood vessels, and the green arrows point to the ducts. **(J)** Weight of ELGs isolated from animals. The values are presented as the means ± SEM, **p < *0.01*, n.s., no significant difference.

**Figure 8:**
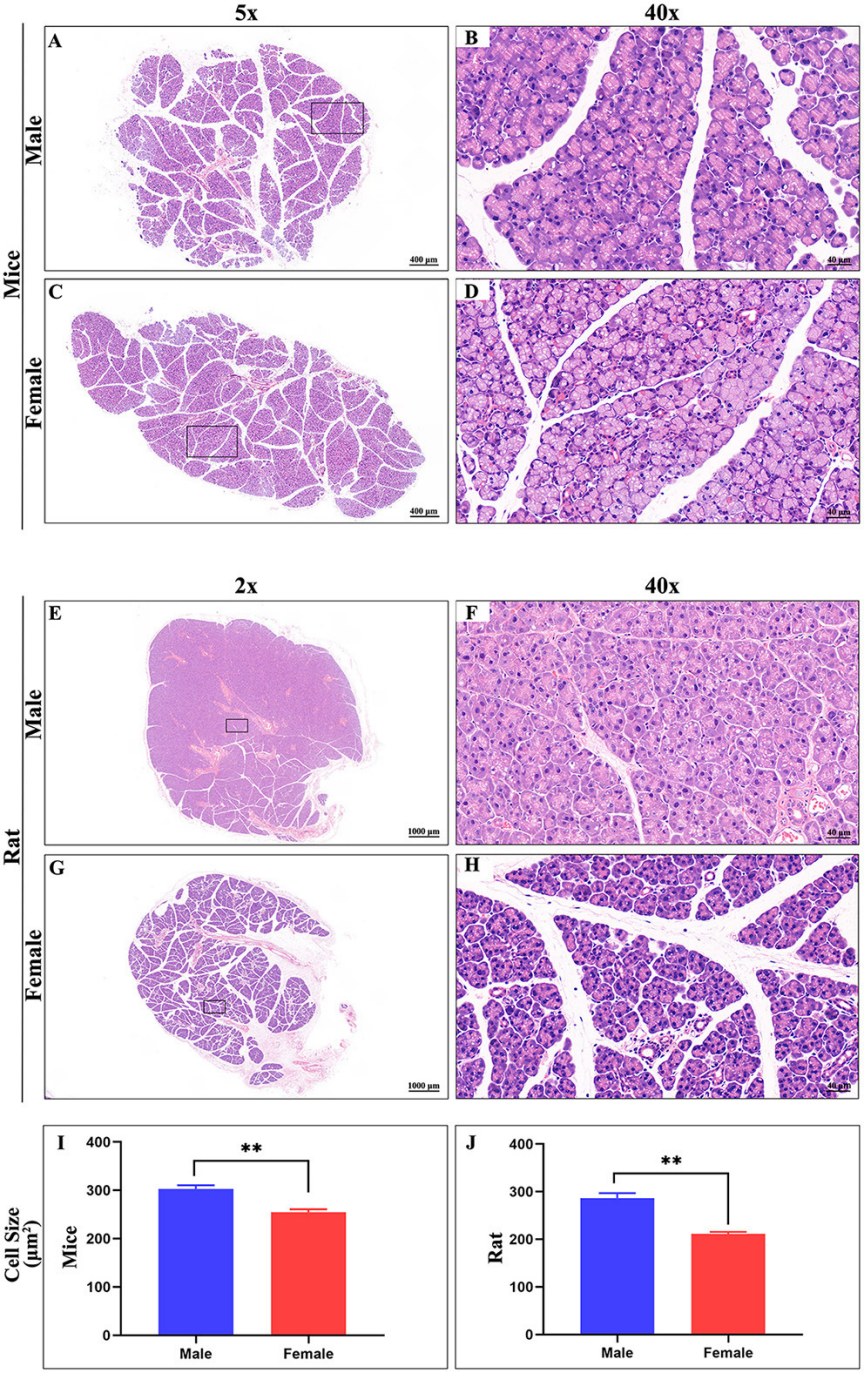
Hematoxylin-eosin stain of ELG. **(A,C)** Representative images of a whole ELG (50 × microscope, scale bar = 400 μm) from mice (**A**, male; **C**, female). **(B,D)** Local amplification (400X microscope, scale bar = 40 μm) of panels on the **(A,C)**, respectively. **(E,G)** Representative images of a whole ELG (20 × microscope, scale bar = 1000 μm) from rats (**A**, male; **C**, female). **(F,H)** Local amplification (400 × microscope, scale bar = 40 μm) of panels on the **(E,G)**, respectively. Average cell size in Hematoxylin-eosin stain images of ELG sections from mice **(I)** and rat **(J)**. The values are presented as the means ± SEM, **p < *0.01*.

The ELG is a flat subcutaneous gland that lies upon the masseter muscles. Mouse ELGs were a bean-shaped milky white tubule-acinar gland encompassed by a fibrous capsule (Figures 7A,B). ELGs were covered and divided by irregular loose connective tissue into 7 lobules, which can be expanded into a snowflake shape (Figures 7C,D). The size of the ELG in male mice was larger than that of the ELGs in female mice. The absolute weight of ELGs was usually greater in males than females (Figure 7J). Rat ELGs were ellipsoid in shape (Figures 7E,F). The glands were also surrounded by connective tissue like mouse ELGs. Scratching the connective tissue around the ELGs, the male rat glands were yellow-brown and close to the color of muscle tissue, while the appearance was pink in female rats (Figures 7E,F). Like the mouse ELGs, the rat ELGs comprised many lobules which were separated from one another by connective tissue. The male glands comprised seven small lobules, whereas the female glands were divided into five large lobes with many small lobules (Figures 7G,H). There is no significant difference in weight between male and female rat ELGs (Figure 7J). Histological analysis showed that the ELGs in either rats or mice were enveloped by a fibrous capsule and lobulated. Each lobule has many acini and intralobular ducts (Figures 8A,C,E,G). The pyramid-shaped acinar cells have a granular and basophilic cytoplasm with a basally located nucleus, with males' acinar cells presenting as larger with wide lumina and nuclear polymorphisms (Figures 8B,D,F,H–J). A T2 structure MRI of the male rat's ELG is shown in Figure 7I.

### Qualitative Analysis of Free Fatty Acids in the ELGs Using the Chemical Isotope Labeling-Assisted Liquid Chromatography-Mass Spectrometry (CIL-LC-MS) Technique

The CIL-LC-MS technique was applied to analyze free fatty acids (FFAs) in the ELGs. A total of 43 potential FFAs were positively identified in the ELGs, as shown in Supplementary Table 4. Among them, there were 13 saturated fatty acids, four monounsaturated fatty acids, four polyunsaturated fatty acids (PUFAs), and three bile acids. By searching the pathway map (e.g., KEGG), we found that some of the fatty acids play an important role in the metabolic pathway and some of them are involved in the biosynthesis of secondary metabolites. It is noteworthy that, except for food digestion roles, bile acids play vital roles in olfactory-mediated behavior, such as conveying general information about other animals in the environment (Doyle and Meeks, 2018). The chemicals that trigger a social response in members of the same species are called pheromones. Given the chemical stability which allows these molecules to play secondary, external roles as chemical messengers after their excretion *via* urine, feces, or other shed substances, many steroids that have been identified as pheromones have solid backgrounds. It has been established that 20 alpha-hydroxycholesterol participates in steroid hormone biosynthesis. Interestingly, isocaproic acid is a metabolite of 20 alpha-hydroxycholesterol (Shimizu et al., 1960). Valproate has been found to affect social behavior in mice (Chapman and Cutler, 1987). In addition to previously known exocrine gland–secreting peptides (ESP1) (Kimoto et al., 2005) and cystatin-related protein 1 (ratCRP1) (Tsunoda et al., 2018), these findings further establish the pheromonal role of the ELGs.

### Analysis of 13C Labeled Metabolites in the TCA Cycle of the ELGs Using the POCE NMR

It is well known that the oxidation of glucose is the main mechanism for energy production. The tricarboxylic acid (TCA) cycle in the mitochondria of ELGs plays an important role in cell metabolism by providing adenosine triphosphate (ATP) and metabolic intermediates for life activities (Martínez-Reyes and Chandel, 2020). The majority of research to date has focused on large molecular proteins and seldom reports on the relevant small molecular metabolites in ELGs. Secretory phospholipase A2 (sPLA2), defensin1, and other bactericidal peptides detected in the acinar cells of ELGs may be protective by resisting foreign microorganisms (Yokoo et al., 2011). An increase in the acute phase of proteins and kininogen mRNA was found in the rat ELGs under the condition of experimental inflammation (Wei et al., 2001). NMR spectroscopy detects specific chemical groups of metabolites through chemical shifts, while the highly sensitive ^1^H observed/^13^C-edited (POCE) NMR technique can disclose a large number of tissue metabolites (Govindaraju et al., 2000), which makes it suitable for exploring the energy metabolites in ELGs (de Graaf et al., 2003).

**Figure 9:**
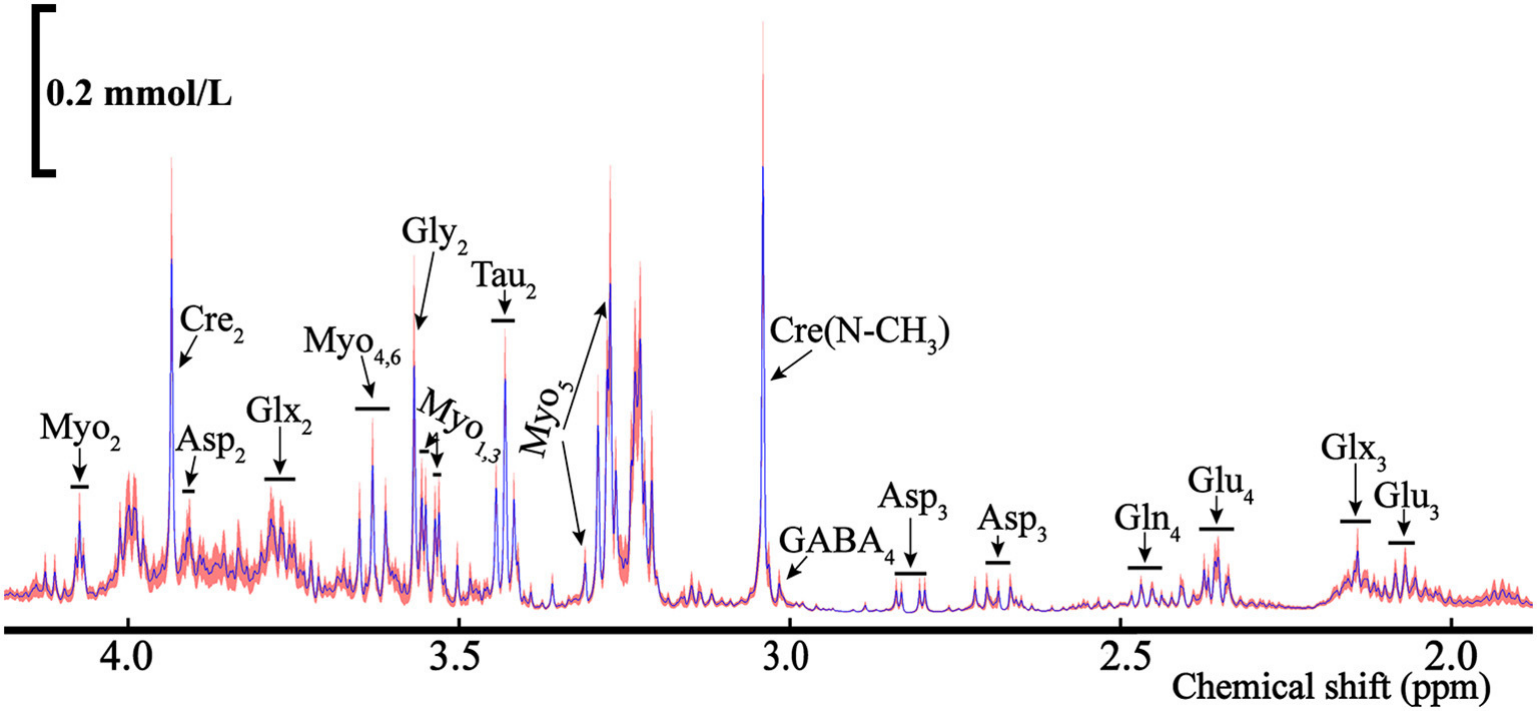
The average NMR spectra for total metabolites (^12^C+^13^C) from the POCE (^1^H observed/^13^C edited) for the rat's ELGs. The blue line represents the average concentration of metabolites, with the red shaded part being the SEM.

The POCE pulse sequence was utilized to explore the metabolic composition of the ELG extracts by distinguishing the types of the metabolites and the total concentration of each substance. NMR spectra acquired by POCE are illustrated in Figure 9, after data processing. [1-^13^C]-glucose enters the TCA cycle during metabolism, marking the carbon position in metabolites produced at different stages *(****reference*** *proton nmr)*. The detected substances included aspartate (Asp), creatine (Cre), γ-aminobutyric acid (GABA), glutamine (Gln), glutamate (Glu), Glx (Glu + Gln), glycine (Gly), myo-inositol (Myo), and Taurine (Tau).

As some metabolites presented in multiple peaks, shown in Figure 9, we selected the pure signal of a certain substance (i.e., Asp_3_) to calculate its concentration *via* integration. The metabolic substances with the least overlapping signal peaks were employed for the concentration calculation as the quantitative analysis of metabolites with mixed signals (i.e., Glu_3_, Gln_4_, etc.) is relatively difficult. The ELGs metabolites and corresponding concentrations in the NMR spectra were calculated and are listed in Table 1. The weighted bilateral ELGs were approximately 0.21 ± 0.04 g. TMSP was used as an internal standard compound to calculate the concentration of ELG metabolites. In different buffers, the chemical shift of the CH_3_ group in TMSP was 0 ppm. The shift of the buffers was different from the chemical shift of various metabolites to avoid overlapping with the peak of the substance tested. The average concentration of Asp_2_ and Glu_4_ extracted from the ELGs were approximately 0.05 ± 0.02 mmol/L and 0.21 ± 0.07 mmol/L respectively, and their contents in different rats were stable (Table 1). The average concentration of Tau_2_ and Cre_2_ were 0.62 mmol/L and 0.39 mmol/L respectively (Table 1).

**Table 1:** Concentration of different metabolites relative to TMSP (means ± SEM) in the rats' ELGs.

**Metabolites**	**Glu_**3**_**	**Glx_**3**_**	**Glu_**4**_**	**Asp_**2**_**	**Cre(N-CH_**3**_)**	**Tau_**2**_**	**Myo_**4**_**	**Glx_**2**_**	**Cre_**2**_**	**Myo_**2**_**
Concentration(mmol/L)	0.04 ± 0.02	0.34 ± 0.08	0.21 ± 0.07	0.05 ± 0.02	0.70 ± 0.17	0.62 ± 0.11	0.25 ± 0.08	0.57 ± 0.06	0.39 ± 0.10	0.17 ± 0.06

## Discussion

This study highlighted the heterogeneity of whole brain innervations to ELGs across the AP axis by PRV based retrograde trans-multi-synaptic system, and the homogeneity of labeling patterns between genders. Due to the nature of PRV labeling, these marked regions work collaboratively to regulate ELGs functions in an indirect manner. While different lobes of ELG injected either PRV531-GFP or PRV724-dsRed showed highly colocalized GFP+ and dsRed+ neurons across the brain, indicating the crosstalk of functional relevance between lobes. Given that there are very limited papers with quantitative analysis, we demonstrated the proportions and distributions of whole brain innervations to the ELGs and quantitatively compared the relative contributions of GFP+ and dsRed+ from the critical brain subregions. In this study, we provided a systematic and quantitative description of whole-brain direct innervations to the ELGs in male and female rats and mice, with statistical evaluation. Qualitative analysis of FFAs and metabolites in the ELGs of male subjects was also undertaken using CIL-LC-MS and NMR techniques.

### Innervation Patterns Across the Whole Brain to ELGs and Their Functional Relevance

ELGs are exocrine glands that lie upon the masseter muscles. In rats and mice, the male glands are generally heavier and bigger than those of females, with no significant difference detected. The ELGs of both mice and rats are capsuled and lobulated across genders. From our HE results, the ELGs in rats and mice are serous tubuloalveolar glands with basally located, round, and variably sized nuclei. Our findings showed that males and females have shared similarities in labeling across the brain regions/subregions with distinctive distribution also present for each gender. The top three sources of labeled neurons were found to be the HY, MB, and STR in males, and HY, MB, and medulla in females. GFP+ was the predominant contributor in all major brain regions and subregions.

ELGs received dominant innervations from the HY along the longitudinal axis of the whole brain, within which the subregion LHA and PSTN showed the two highest numbers of labeled neurons. HY has long been considered as a master homeostatic regulator in modulating life crucial activities such as growth and reproductive behaviors, stress response, along with social and emotional behaviors (Burbridge et al., 2016). Our statistical analysis revealed that almost half of the labeled neurons from the HY were located in the LHA, which is one of the larger and more heterogeneous regions of the HY that comprise three key cell populations, namely the melanin-concentrating hormone (MCH), orexin/hypocretin, and GABAergic subpopulations, to mediate sleep-wake cycle, energy balance, reward and motivated behaviors, feeding behavior, and arousal (Arrigoni et al., 2019). Recent studies have shown that genetic deletion of LHA MCH receptors (MCHR1) in mice produces social deficits and mood disruption (Sanathara et al., 2021). LHA orexin has been proven to promote feeding and increase energy expenditure in mice *via* intra-hypothalamic connections to the paraventricular hypothalamic nucleus (PVH), dorsomedial hypothalamic nucleus (DMH), and within the LHA (Arrigoni et al., 2019). The second contributor of labeled neurons was PSTN, which has been extensively explored in terms of its role in regulating feeding behavior and potentially cardiovascular functions (Goto and Swanson, 2004; Ciriello et al., 2008; Zhu et al., 2012; Chometton et al., 2016; Barbier et al., 2017, 2020). The landmark work of Goto and Swanson a decade ago led to the general impression about PSTN projections by using the anterograde tracer *Phaseolus vulgaris* leucoagglutinin (PHAL), which reveals the densest input to several gustatory brain centers such as the rostral nucleus of the solitary tract (NTS), the gustatory area of the insular cortex or the superior salivatory nucleus (Goto and Swanson, 2004; Chometton et al., 2016). They also proposed PSTN-mastered gustatory and viscerosensory information *via* projections to the NTS and PB, along with parasympathetic responses associated with feeding and cardiovascular functions (Goto and Swanson, 2004). There is continuing evidence revealing the role of PSTN in feeding behavior, which was potentially achieved through the control of appetite (Chometton et al., 2016; Barbier et al., 2017, 2020). It is interesting to note that these clues all advocated that PSTN has specific and robust responses associated with the ingestion of palatable food, which might be vital for survival in conflicting situations of potentially noxious or previously unknown food sources (Chometton et al., 2016; Barbier et al., 2020). The surrounding areas of PSTN including STN and ZI, which according to our results have less densely labeled neurons, also show highly relevant functional capacity in emotional information processing from research outputs (Buot et al., 2013; Zhang and Van Den Pol, 2017). Previous electrophysiological studies in animals and humans have suggested that STN neuronal activity is modified not only by motor effects but also in relation to relevant emotions and behaviors, such as reward prediction and obtainment (Teagarden and Rebec, 2007; Lardeux et al., 2009; Sauleau et al., 2009). ZI has also been shown to modulate energy homeostasis and food consumption *via* its powerful inhibitory GABA projections (Zhang and Van Den Pol, 2017). Only one subregionally significant difference in labeling was documented in the region called LPO, in which females have a substantially higher number of labeled neurons than males. LPO is an anterior hypothalamic region involved in reward behavior and it is functionally connected to the ventral tegmental area (VTA) (Gordon-Fennell et al., 2020). It has been proposed by previous studies that stimulating LPO reinstated cocaine and sucrose-seeking behavior *via* inhibition of VTA GABA neurons and enhancement of VTA dopamine neurons (Gordon-Fennell et al., 2020). The gender-based disparities of labeling patterns within the LPO indicate the sex-specific functions of ELGs in females, which might be facilitated by female-exclusive pheromones or hormones. Except for the above subregions of the HY, we also found sparsely labeled neurons located in the MPO, MPN, DMH, VMH, and PH, all of which have been suggested to support several non-negligible functions in social behavior and emotional regulation (DiMicco et al., 2002; Kunwar et al., 2015; Maejima et al., 2015; McHenry et al., 2017; Iovino et al., 2019). Taken together, it could be speculated that heavy hypothalamic innervations to the ELGs may facilitate ELG-regulated pheromone release in response to external stimuli and eventually modify the social behaviors of anilmals.

Male mice were reported to have a significantly higher number of labeled neurons in the OLF, which is the region that detects chemical cues and generates adaptive emotional responses (Kondoh et al., 2016). Our results demonstrated that the OLF subregions TR and Pir offered dominant innervations to the ELGs. TR, which comprises glutamate releasing principal neurons and GABA-releasing interneurons, has been identified to induce stress hormone responses to volatile predator odors and mimic an instinctive fear response in mice (Kondoh et al., 2016). Pir is a key brain area responsible for not only olfactory information coding but also higher-order associative functions including emotional visual information processing, as indicated by previous functional magnetic resonance imaging (fMRI) (Schulze et al., 2017). Above emotion-related functions governed by the OLF and its associated subregions also index to the physiological and functional connectivity to the ELGs as the consequence of intense innervations.

Our tracing studies showed that the ELGs were also innervated heavily by the STR, which is a critical component of voluntary motor control, reward perception, and cognition (Báez-Mendoza and Schultz, 2013). Two vital sources of the labeled neurons within the STR in males and females were the CEA and MEA, both of which are functionally similar nuclei that comprise over 90% GABAergic neurons (Šimić et al., 2021). Previous animal studies have suggested that olfactory information received by the main and accessory olfactory system will merge into the HY and MEA to activate the release of oxytocin neurons for inter-male social interactions (Onaka et al., 2012). The involvement of MEA by controlling the release of oxytocin in response to social interaction is further proven by MEA lesion, which consequently impairs social interaction (Onaka et al., 2012). The role of CEA in mediating fear- and anxiety-related behavioral and endocrine responses in the primate has been examined by either asymmetric lesions in CEA or bilateral CEA destruction (Kalin et al., 2004). Bilaterally lesioned monkeys displayed significantly less fear-related behaviors when exposed to a snake, less freezing behavior when confronted by a human intruder, and decreased levels of CSF corticotrophin-releasing factor (CRF), all of which support the role of CEA in facilitating emotional and behavioral responses associated with fear and anxiety (Kalin et al., 2004; Phelps and LeDoux, 2005). The complex interplay within the subregions of STR along with between the STR and other emotional-related brain regions such as the HY have provided a foundation on which to explore the representation of emotional modulation by the ELGs.

Albeit no statistically significant differences were detected in pons while normalized, which was related to the whole brain labeled neurons, the subregion PBmm in male mice demonstrated a substantially higher number of labeled neurons than female mice when a regional statistical analysis was conducted. Our results displayed that the top three sources of labeled neurons in males came from PBI, PBmm, and PRNc, whereas signals in females mainly arose from PRNc, PBI, and PBmm. The pons is the portion of the brainstem that relays information about respiration, motor functions, eye movement, and sensation (Zimmer et al., 2020). Aligning with previously mentioned studies of CEA-regulated emotional responses (Kalin et al., 2004), the CRF is also present in the PRNc to facilitate its function in fear and anxiety. A classical study has supported the role of PRNc in mediating the expression of fear-potentiated startle in the rat, which was potentially achieved by the interconnected working network with the CEA for CRF secretion (Fendt, 1997). The PB can be divided along the superior cerebellar peduncle into three main parts: PBI, PBmm, and PBKF, all of which showed a substantial quantity of labeled neurons from our results (Fulwiler and Saper, 1984). Several pioneer studies have outlined that the PBI is vital in transmitting a broad range of sensory information such as feeding and pain to the forebrain (Le May et al., 2021), the PBKF is mainly involved in breathing rhythmogenesis (Yang et al., 2020), and the PBmm plays an important role in controlling wakefulness and behavioral arousal (Xu et al., 2021). All the functional capacities of PB subregions mentioned above and their heavy innervation to ELGs may facilitate the formation of associations between external stimuli and behavior outcomes, which also underlies the *sin qua non* role of ELGs in social behaviors.

### Analysis of FFAs Further Support the Important Role of ELGs in Social Communication

In most mammals, chemical cues called pheromones have emerged as the predominant “language” for communicating social and sexual information (Liberles, 2014). These pheromones are released by individuals, often chemically unrelated, and are contained in body fluids like urine, sweat, specialized exocrine glands, and the mucous secretions of genitals. Although numerous studies have suggested that pheromones originate from urine, recent studies have highlighted the importance of lacrimal proteins for social communication (Liberles, 2014). For example, the lacrimal protein ESP1 has an important role as a sex pheromone that effectively enhances female sexual receptive behavior (Haga et al., 2010). The ratCRP1 isolated from the ELG, induced stopping behavior in female rats (Tsunoda et al., 2018). These observations demonstrate the important role of ELGs in secreting pheromones. Numerous studies have suggested that pheromones display tremendous structural diversity and include volatile organic compounds, fatty acids, and proteins (Wyatt, 2017). To find fatty acids that can be used as pheromones, we used the CIL-LC-MS technique to analyze free fatty acids in the ELGs and compared positively identified fatty acids with pheromones previously defined. In total, 3 bile acids (ursocholic acid, isoursodeoxycholic acid, and 7-ketodeoxycholic acid) were identified in ELGs, and they have been potentially acted as pheromonal cues. Steroids are important pheromones in most mammalian species, especially steroid hormones (Doyle and Meeks, 2018). 20 alpha-hydroxycholesterol were involved in the steroid hormone biosynthesis and isocaproic acid is a metabolite of 20 alpha-hydroxycholesterol (Shimizu et al., 1960). Previous investigations have shown that valproate affects social behavior in mice (Chapman and Cutler, 1987). All these previous defined fatty acid related pheromones mentioned above further support the important role of ELGs in social communication.

### The Relevance Between the ELGs' Energy Metabolism and the Brain From Metabolic Analysis

As the energy metabolism of ELGs is poorly mapped, we conducted NMR metabolic kinetics to detect and quantitatively analyze the metabolites in rat ELGs. Astrocytes Glu-Gln cycle and Glu-GABA cycle exist between GABAergic neurons and glutamatergic neurons (Guo et al., 2021). The labeled carbon in Glu_4_ is transferred to GABA, after which the GABA and Glu are absorbed by astrocytes (Guo et al., 2021). Previous studies have illustrated that the removal of bilateral ELGs in mice induced moderate to severe dry eye, and the removal of an ipsilateral ELG resulted in increased anxiety in female mice, which suggested that neurotransmitter metabolism disorder in ELGs may underlie some mental illnesses (Mecum et al., 2020). Comparing the NMR spectra of brain metabolism, the metabolites in the ELGs were lack of the N-acetylaspartate (NAA) (Navarro et al., 2008), suggesting the discrepancy between the mechanism of energy metabolism in the ELGs and the brain. Different concentrations of ^13^C-labeled Glu detected at different positions may be the result of multiple peaks folding in the integrated area, or the metabolites were not completely extracted from the ELGs. Although there might be some limitations in our research, this study on the metabolites of ELGs is of great reference value for subsequent investigations of physiological functions and metabolic mechanisms.

## Conclusion

The present study elucidated whole-brain direct innervations to ELGs in both genders of rats and mice, undertaking comprehensive histological visualization together with a detailed analysis of the FFAs secreted from the ELGs. Although the innervation patterns are similar for males and females in the most major brain regions along the AP axis, they are diverse for subregional labeling distribution. These findings indicate that ELGs integrate extensive innervations from numerous brain regions in the whole brain range and that males and females are innervated differently by the HY, OLF, and STR, which may contribute to further study of the diverse functions of ELGs. The detailed histological results between rats and mice of both genders undertaken in the present study also provide a structural basis for elaborating the functional relevance of ELGs. Moreover, analysis of FFAs from the ELGs revealed several important FFAs, such as ursocholic acid, isoursodeoxycholic acid, 7-ketodeoxycholic acid, and valproate, which play a central role in guiding behavioral and physiological responses to social reactions.

## Data Availability Statement

The original contributions presented in the study are included in the article/Supplementary Material, further inquiries can be directed to the corresponding author/s.

## Ethics Statement

The animal study was reviewed and approved by the Animal Care and Use Committee at Innovation Academy for Precision Measurement Science and Technology, Chinese Academy of Sciences.

## Author Contributions

YL and FX designed the experiments. YZ, ML, ZG, YW, TH, and YL conducted experiments and analyzed data. YZ, ML, ZG, YL, and FX wrote the manuscript.

## Funding

The work was supported by the National Natural Science Foundation of China (31800876, 31830035, and 21921004), the Key-Area Research and Development Program of Guangdong Province (2018B030331001), the Strategic Priority Research Program of the Chinese Academy of Sciences (XDB32030200), and Shenzhen Key Laboratory of Viral Vectors for Biomedicine (ZDSYS20200811142401005).

## Conflict of Interest

The authors declare that the research was conducted in the absence of any commercial or financial relationships that could be construed as a potential conflict of interest.

## Publisher's Note

All claims expressed in this article are solely those of the authors and do not necessarily represent those of their affiliated organizations, or those of the publisher, the editors and the reviewers. Any product that may be evaluated in this article, or claim that may be made by its manufacturer, is not guaranteed or endorsed by the publisher.
