# Supplementary material for: Whole Brain Mapping of Neurons Innervating Extraorbital Lacrimal Glands in Mice and Rats of Both Genders
Source: Front Neural Circuits. 2021 Oct 29;15:768125. doi: 10.3389/fncir.2021.768125 (PMC8585839; doi:10.3389/fncir.2021.768125)
Supplement: Supplementary file 1 [file Data_Sheet_1.docx]

**Whole brain mapping of neurons innervating extraorbital lacrimal glands in mice and rats of both genders**

Ying Zhai^a,f,1^, Min Li^a,g,h,1^, Zhu Gui^a^, Yeli Wang^a,i^, Ting Hu^a^, Yue Liu^a,*^, Fuqiang Xu^a,b,c,d,e^,^*^

a. State Key Laboratory of Magnetic Resonance and Atomic and Molecular Physics, Key Laboratory of Magnetic Resonance in Biological Systems, Wuhan Center for Magnetic Resonance, Innovation Academy for Precision Measurement Science and Technology, Chinese Academy of Sciences, Wuhan 430071, China.

b. The Brain Cognition and Brain Disease Institute (BCBDI), Shenzhen Key Laboratory of Viral Vectors for Biomedicine ,Shenzhen Institute of Advanced Technology, Chinese Academy of Sciences; Shenzhen-Hong Kong Institute of Brain Science-Shenzhen Fundamental Research Institutions, NMPA Key Laboratory for Research and Evaluation of Viral Vector Technology in Cell and Gene Therapy Medicinal Products, Shenzhen, Key Laboratory of Quality Control Technology for Virus-Based Therapeutics, Guangdong Provincial Medical Products Administration, Shenzhen, 518055, China

c. Center for Excellence in Brain Science and Intelligence Technology, Chinese Academy of Sciences, Shanghai 200031, China.

d. University of Chinese Academy of Sciences, Beijing 100049, PR China.

e. Wuhan National Laboratory for Optoelectronics, Huazhong University of Science and Technology, Wuhan 430074, China.

f. Centre for Brain Research, Department of Anatomy and Medical Imaging, Faculty of Medical and Health Sciences, University of Auckland, Auckland 1023, New Zealand

g. Basic Medical Laboratory, General Hospital of Central Theater Command, Wuhan 430070, China

h. Hubei Key Laboratory of Central Nervous System Tumor and Intervention, Wuhan 430070, China

i. College of Life Sciences, Wuhan University, Wuhan 430072, China

^1:^ These authors contributed equally to this work

*** Correspondence:**Corresponding Author
**Yue Liu**: E-mail: liuyue@apm.ac.cn;

**Fuqiang Xu**: E-mail: fuqiang.xu@wipm.ac.cn; Tel: +86-27-87197091; Fax: +86-27-87199543.

***
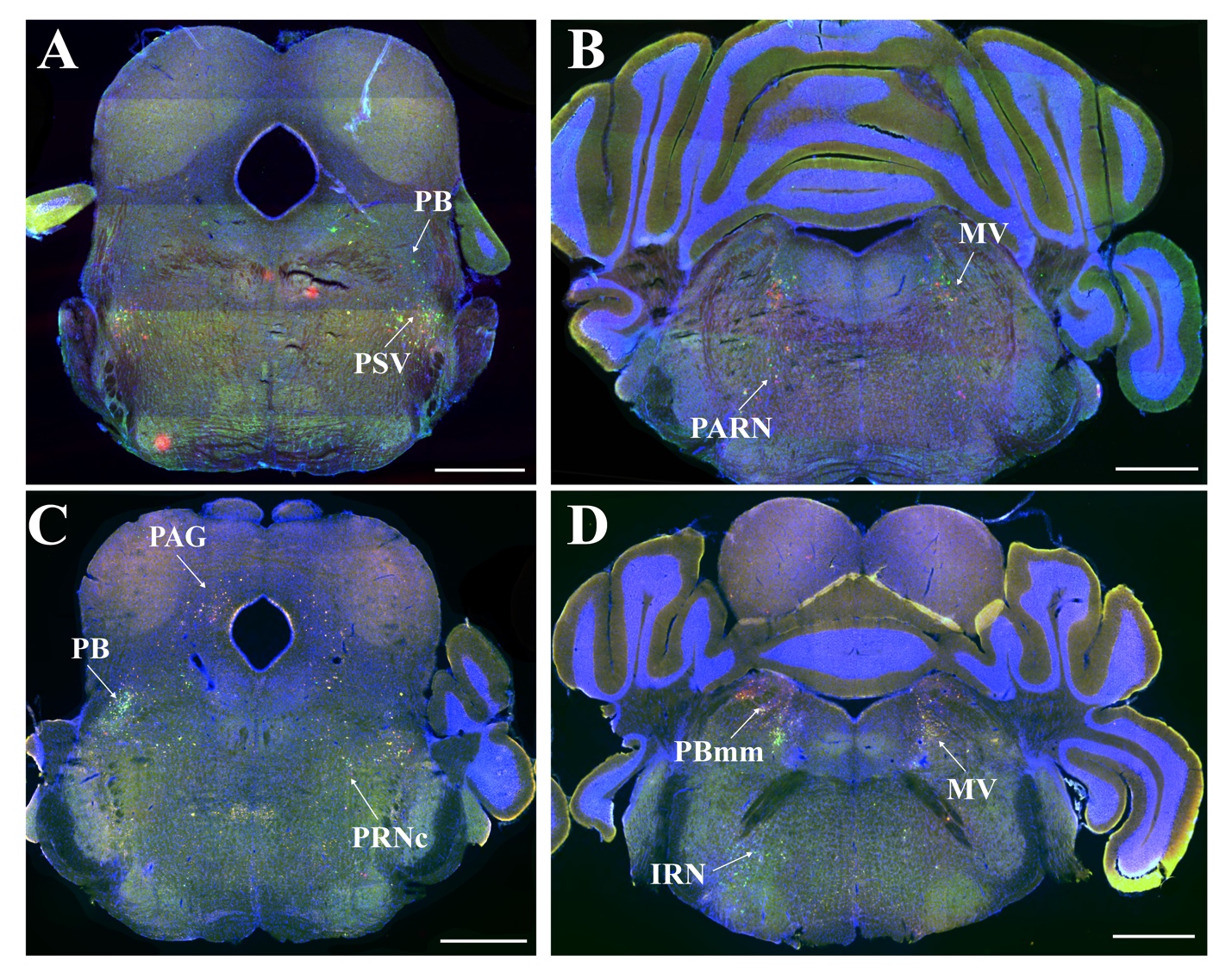
Supplementary material***

**Figure 1 |** Distribution patterns of labelled neurons after 2.5d PRV transmission. (A,B) Representative images showing labelled neurons from male mouse with PRV injection on ELGs. Scale bar: 1mm. (C,D) Representative images showing labelled neurons from female mouse with PRV injection on ELGs. Scale bar: 1mm. Green: GFP; red: dsRed; blue: DAPI. PB: parabrachial nucleus; PBmm: parabrachial nucleus, medial division, medial medial part; PSV: principal sensory nucleus of the trigeminal; MV: medial vestibular nucleus; PARN: parvicellular reticular nucleus; PRNc: potine reticular nucleus, caudal part.

**Supplementary Table 1: Abbreviations**

| **Abbreviation** | **Definition** |
| --- | --- |
| **Isocortex**  SSp  AI (d/v/p)  PL  MO (p/s)  ECT  PERI  AUD (d/v/p)  **OLF**  Pir  TR  TT (d/v)  **STR**  LS(r/c/v)  CEA  MEA  ACB  **PAL**  BST (a/p)  SI  **HY**  LPO  LHA  MPO  MPN  DMH  VMH  PSTN  STN  PH  ZI  **CTXsp**  BLAa  BLAp  BMAp  **MB**  PAG  MRN  VTA  SCm  **Pons**  PRNr  PRNc  PBl  PBmm  PBKF  **Medulla**  PARN  MARN  PGRNl  IRN  GRN  VII  MV  ACN  CMPI  CIL-LC-MS  EA  FA  PBS  RT  TEA  FFAs  TCA  ATP  sPLA2  POCE  NMR  Myo  Cre  Asp  Glx  Gly  Glu  Gln  Tau  GABA  TMSP  NAA | Primary somatosensory area  Agranular insular area  Prelimbic area  Somatomotor areas  Ectorhinal area  Perirhinal area  Auditory areas  **Olfactory areas**  Piriform area  Post-piriform transition area  Taenia tecta  **Striatum**  Lateral septal nucleus  Central amygdalar nucleus  Medial amygdalar nucleus  Nucleus accumbens  **Pallidum**  Bed nuclei of the stria terminalis  Substantia innominata  **Hypothalamus**  Lateral preoptic area  Lateral hypothalamic area  Medial preoptic area  Medial preoptic nucleus  Dorsomedial nucleus of the hypothalamus  Ventromedial nucleus of the hypothalamus  Parasubthalamic nucleus  Subthalamic nucleus  Posterior hypothalamic nucleus  Zona incerta  **Cortical subplate**  Basolateral amygdalar nucleus, anterior  Basolateral amygdalar nucleus, posterior  Basomedial amygdalar nucleus  **Midbrain**  Periaqueductal gray  Midbrain reticular nucleus  Ventral tegmental area  Superior colliculus, motor related  Pontine reticular nucleus  Pontine reticular nucleus, caudal part  Parabrachial nucleus, lateral division  Parabrachial nucleus, medial division, medial medial part  Koelliker-Fuse subnucleus  Parvicellular reticular nucleus  Magnocellular reticular nucleus  Paragigantocellular reticular nucleus, lateral part  Intermediate reticular nucleus  Gigantocellular reticular nucleus  Facial motor nucleus  Medial vestibular nucleus  Acetonitrile  2-chloro-1-methylpyridinium iodide  Chemical isotope labelling-assisted liquid chromatography-mass spectrometry  Ethyl acetate  Formic acid  Phosphate-buffered saline  Retention time  Triethylamine  Free fatty acids  Tricarboxylic acid  Adenosine triphosphate  Secretory phospholipase A2  ^1^H observed/^13^C-edited  Nuclear magnetic resonance  Myo-inositol  Creatine  Aspartate  Glutamine + glutamate  Glycine  Glutamate  Glutamine  Taurine  γ-aminobutyric acid  3-(Trimethylsilyl) propionic-2,2,3,3-d4 acid sodium salt  N-acetylaspartate |

**Supplementary Table 2:**

**Quantification of total input cells across the whole brain for males**

|  | 1#mouse | 2#mouse | 3#mouse |
| --- | --- | --- | --- |
| DsRed+ | 1910 | 959 | 2047 |
| GFP+ | 5216 | 4195 | 1330 |

**Quantification of total input cells across the whole brain for females**

|  | 1#mouse | 2#mouse | 3#mouse |
| --- | --- | --- | --- |
| DsRed+ | 587 | 3727 | 4263 |
| GFP+ | 2617 | 11267 | 5090 |

**Supplementary Table 3: Statistical analysis**

| Figure | Target | Data values | P values | Methods |
| --- | --- | --- | --- | --- |
| 2B | Isocortex | 4.85%$\pm$1.79% for males vs females, 4.38%$\pm$ 1.14% | 0.829 | T-tests |
|  | OLF | 4.01%$\pm$0.89% for males vs females, 1.54%$\pm$0.53% | **0.037** | T-tests |
|  | STR | 16.79%$\pm$1.44% for males vs females, 9.85%$\pm$2.26% | **0.027** | T-tests |
|  | PAL | 1.68%$\pm$1.04% for males vs females, 3.49%$\pm$0.84% | 0.207 | T-tests |
|  | HY | 20.30%$\pm$1.95% for males vs females, 29.93%$\pm$1.51% | **0.003** | T-tests |
|  | CTXsp | 5.56%$\pm$1.93% for males vs females,7.58%$\pm$3.21% | 0.600 | T-tests |
|  | MB | 18.51%$\pm$3.72% for males vs females, 13.78%$\pm$1.11% | 0.251 | T-tests |
|  | Pons | 11.87%$\pm$1.56% for males vs females, 8.19%$\pm$1.60% | 0.130 | T-tests |
|  | Medulla | 16.41%$\pm$4.90% for males vs females, 21.28%$\pm$4.74% | 0.492 | T-tests |
|  |  |  |  |  |
| 2C (GFP+) | Isocortex | 5.10%$\pm$2.77% for males vs females, 4.50%$\pm$2.16% | 0.873 | T-tests |
|  | OLF | 4.99%$\pm$1.39% for males vs females, 2.16%$\pm$0.82% | 0.155 | T-tests |
|  | STR | 16.61%$\pm$2.67% for males vs females, 10.31%$\pm$3.44% | 0.222 | T-tests |
|  | PAL | 1.53%$\pm$1.49% for males vs females, 3.04%$\pm$0.57% | 0.398 | T-tests |
|  | HY | 18.52%$\pm$2.5% for males vs females, 29.43%$\pm$1.93% | **0.026** | T-tests |
|  | CTXsp | 5.69%$\pm$2.83% for males vs females, 9.33%$\pm$5.97% | 0.611 | T-tests |
|  | MB | 20.14%$\pm$7.04% for males vs females, 13.71%$\pm$1.32% | 0.420 | T-tests |
|  | Pons | 13.70%$\pm$2.09% for males vs females, 8.29%$\pm$3.33% | 0.271 | T-tests |
|  | Medulla | 13.72%$\pm$6.09% for males vs females, 19.22%$\pm$8.63% | 0.630 | T-tests |
|  |  |  |  |  |
| 2C (dsRed+) | Isocortex | 4.60%$\pm$2.9% for males vs females, 4.25%$\pm$1.36% | 0.919 | T-tests |
|  | OLF | 3.04%$\pm$1.02% for males vs females, 0.91%$\pm$0.57% | 0.144 | T-tests |
|  | STR | 16.97%$\pm$1.79% for males vs females, 9.38%$\pm$3.66% | 0.136 | T-tests |
|  | PAL | 1.84%$\pm$1.79% for males vs females, 3.94%$\pm$1.72% | 0.447 | T-tests |
|  | HY | 22.09%$\pm$3.09% for males vs females, 30.42%$\pm$2.73% | 0.113 | T-tests |
|  | CTXsp | 5.42%$\pm$3.24% for males vs females, 5.83%$\pm$3.60% | 0.936 | T-tests |
|  | MB | 16.88%$\pm$4.12% for males vs females,13.84%$\pm$2.11% | 0.547 | T-tests |
|  | Pons | 10.05%$\pm$2.11% for males vs females, 8.09%$\pm$1.30% | 0.473 | T-tests |
|  | Medulla | 19.11%$\pm$8.70% for males vs females, 23.33%$\pm$5.82% | 0.707 | T-tests |
|  |  |  |  |  |
| 2D | SSp | 0.09%$\pm$0.07% for males vs females, 0.47%$\pm$0.19% | 0.077 | T-tests |
|  | AI | 3.04%$\pm$1.30% for males vs females, 1.16%$\pm$0.38% | 0.196 | T-tests |
|  | PL | 0.03%$\pm$0.02% for males vs females, 0.11%$\pm$0.05% | 0.137 | T-tests |
|  | MO | 0.25%$\pm$0.15% for males vs females, 0.39%$\pm$0.20% | 0.572 | T-tests |
|  | ECT | 0.38%$\pm$0.26% for males vs females, 1.04%$\pm$0.34% | 0.153 | T-tests |
|  | PERI | 1.07%$\pm$0.46% for males vs females, 0.85%$\pm$0.31% | 0.697 | T-tests |
|  | AUD | 0 for males vs females, 0.36%$\pm$0.21% | 0.113 | T-tests |
|  | Pir | 0.94%$\pm$0.35% for males vs females, 0.76%$\pm$0.17% | 0.658 | T-tests |
|  | TR | 3.03%$\pm$1.08% for males vs females, 0.50%$\pm$0.37% | **0.052** | T-tests |
|  | TT | 0.04%$\pm$0.02% for males vs females, 0.27%$\pm$0.13% | 0.124 | T-tests |
|  | LS | 0.76%$\pm$0.35% for males vs females, 1.00%$\pm$0.19% | 0.554 | T-tests |
|  | CEA | 11.16%$\pm$1.72% for males vs females, 7.16%$\pm$2.59% | 0.227 | T-tests |
|  | MEA | 4.65%$\pm$2.59% for males vs females, 1.64%$\pm$0.37% | 0.276 | T-tests |
|  | ACB | 0.22%$\pm$0.14% for males vs females, 0.05%$\pm$0.03% | 0.281 | T-tests |
|  | BST | 1.50%$\pm$0.94% for males vs females, 3.17%$\pm$0.87% | 0.223 | T-tests |
|  | SI | 0.18%$\pm$0.13% for males vs females, 0.32%$\pm$0.12% | 0.444 | T-tests |
|  | LPO | 0.09%$\pm$0.05% for males vs females, 0.87%$\pm$0.29% | **0.025** | T-tests |
|  | LHA | 8.31%$\pm$2.31% for males vs females,9.83 %$\pm$1.04% | 0.561 | T-tests |
|  | MPO | 1.18%$\pm$0.53% for males vs females, 2.07%$\pm$0.27% | 0.162 | T-tests |
|  | MPN | 0.77%$\pm$0.56% for males vs females, 1.41%$\pm$0.48% | 0.403 | T-tests |
|  | DMH | 1.33%$\pm$0.45% for males vs females, 1.54%$\pm$0.26% | 0.691 | T-tests |
|  | VMH | 0.33%$\pm$0.22% for males vs females, 1.09%$\pm$0.44% | 0.152 | T-tests |
|  | PSTN | 4.39%$\pm$1.84% for males vs females, 9.31%$\pm$0.96% | **0.040** | T-tests |
|  | STN | 0.70%$\pm$0.24% for males vs females, 0.43%$\pm$0.20% | 0.402 | T-tests |
|  | PH | 1.49%$\pm$0.49% for males vs females, 1.75%$\pm$0.47% | 0.716 | T-tests |
|  | ZI | 1.70%$\pm$1.05% for males vs females, 1.62%$\pm$0.72% | 0.946 | T-tests |
|  | BLAa | 5.18%$\pm$2.04% for males vs females, 3.66%$\pm$1.47% | 0.559 | T-tests |
|  | BLAp | 0.25%$\pm$0.18% for males vs females, 1.11%$\pm$0.43% | 0.093 | T-tests |
|  | BMAp | 0.13%$\pm$0.08% for males vs females,2.82 %$\pm$1.57% | 0.118 | T-tests |
|  | PAG | 12.42%$\pm$2.21% for males vs females, 9.64%$\pm$0.65% | 0.255 | T-tests |
|  | MRN | 5.23%$\pm$1.31% for males vs females, 3.35%$\pm$0.42% | 0.202 | T-tests |
|  | VTA | 0.78%$\pm$0.42% for males vs females, 0.49%$\pm$0.31% | 0.575 | T-tests |
|  | SCm | 0.07%$\pm$0.05% for males vs females, 0.30%$\pm$0.13% | 0.140 | T-tests |
|  | PRNr | 0.32%$\pm$0.11% for males vs females, 0.67%$\pm$0.25% | 0.225 | T-tests |
|  | PRNc | 2.93%$\pm$0.65% for males vs females, 3.83%$\pm$1.38% | 0.570 | T-tests |
|  | PBI | 4.29%$\pm$0.66% for males vs females, 2.45%$\pm$0.74% | 0.092 | T-tests |
|  | PBmm | 3.93%$\pm$1.08% for males vs females, 0.80%$\pm$0.59% | **0.029** | T-tests |
|  | PBKF | 0.41%$\pm$0.29% for males vs females, 0.44%$\pm$0.28% | 0.962 | T-tests |
|  | PARN | 3.55%$\pm$1.56% for males vs females, 4.20%$\pm$1.23% | 0.750 | T-tests |
|  | MARN | 2.93%$\pm$1.03% for males vs females, 6.48%$\pm$1.59% | 0.089 | T-tests |
|  | PGRNI | 0.18%$\pm$0.11% for males vs females, 1.95%$\pm$1.33% | 0.216 | T-tests |
|  | IRN | 4.31%$\pm$1.35% for males vs females, 3.79%$\pm$1.12% | 0.774 | T-tests |
|  | GRN | 0.69%$\pm$0.17% for males vs females, 2.21%$\pm$0.81% | 0.099 | T-tests |
|  | VII | 3.01%$\pm$1.20% for males vs females, 1.16%$\pm$0.62% | 0.201 | T-tests |
|  | MV | 1.75%$\pm$1.14% for males vs females, 1.48%$\pm$0.78% | 0.850 | T-tests |
|  |  |  |  |  |
| 3D | LPO | 0.41%$\pm$0.35% for males vs females, 3.79%$\pm$1.01% | **0.033** | T-tests |
|  | LHA | 41.99%$\pm$% for males vs females, 34.53%$\pm$3.37% | 0.719 | T-tests |
|  | MPO | 5.94%$\pm$3.52% for males vs females, 6.96%$\pm$1.17% | 0.797 | T-tests |
|  | MPN | 4.05%$\pm$3.16% for males vs females, 4.82%$\pm$2.51% | 0.859 | T-tests |
|  | DMH | 7.17%$\pm$3.78% for males vs females, 5.22%$\pm$0.94% | 0.641 | T-tests |
|  | VMH | 1.56%$\pm$1.56% for males vs females, 3.89%$\pm$2.51% | 0.476 | T-tests |
|  | PSTN | 20.48%$\pm$11.90% for males vs females, 29.29%$\pm$2.50% | 0.254 | T-tests |
|  | STN | 3.95%$\pm$2.20% for males vs females, 1.37%$\pm$0.10% | 0.345 | T-tests |
|  | PH | 6.58%$\pm$3.11% for males vs females, 5.55%$\pm$2.21% | 0.801 | T-tests |
|  | ZI | 7.87%$\pm$7.57% for males vs females, 5.36%$\pm$3.44% | 0.778 | T-tests |
|  |  |  |  |  |
| 4D | Pir | 37.73%$\pm$28.31% for males vs females, 59.57%$\pm$18.97% | 0.557 | T-tests |
|  | TR | 60.10%$\pm$28.86% for males vs females, 26.54%$\pm$15.96% | 0.366 | T-tests |
|  | TT | 2.85%$\pm$0.48% for males vs females, 13.89%$\pm$6.46% | 0.163 | T-test |
|  |  |  |  |  |
| 5D | LS | 4.48%$\pm$2.00% for males vs females, 11.85%$\pm$6.08% | 0.313 | T-tests |
|  | CEA | 72.49%$\pm$16.02% for males vs females, 64.36%$\pm$14.07% | 0.723 | T-tests |
|  | MEA | 21.90%$\pm$18.22% for males vs females, 23.06%$\pm$9.86% | 0.958 | T-tests |
|  | ACB | 1.14%$\pm$0.94% for males vs females, 0.73%$\pm$0.31% | 0.702 | T-tests |
|  |  |  |  |  |
| 6D | PRNr | 2.49%$\pm$1.05% for males vs females, 12.29%$\pm$6.62% | 0.217 | T-tests |
|  | PRNc | 26.99%$\pm$9.25% for males vs females, 33.59%$\pm$18.22% | 0.758 | T-tests |
|  | PBI | 33.99%$\pm$1.95% for males vs females, 29.75%$\pm$4.68% | 0.451 | T-tests |
|  | PBmm | 30.85%$\pm$8.25% for males vs females, 19.60%$\pm$17.70% | 0.595 | T-tests |
|  | PBKF | 5.68%$\pm$5.37% for males vs females, 4.77%$\pm$4.21% | 0.900 | T-tests |

**Supplementary Table 4:** **Content of the detected potential fatty acids in ELG**

| No. | Time | RI | m/z (DMED-labeled) | m/z (d4-DMED-labeled) | Intensity (cps) d4-DMED-labeled | Formula (DMED-labeled) | Formula (unlabeled) | Molecular weight | Name | CASNo. | KEGG ID |
| --- | --- | --- | --- | --- | --- | --- | --- | --- | --- | --- | --- |
| 1 | 3.38931 | 600.3782 | 187.1807 | 191.2058 | 88328893.18 | C10H23ON2 | C6H12O2 | 116.0837 | Isocaproic acid | 646-07-1 | - |
| 2 | 3.55929 | 611.7983 | 199.1808 | 203.2058 | 59488947.13 | C11H23ON2 | C7H12O2 | 128.0837 | Cyclohexanecarboxylic acid | 98-89-5 | C09822 |
| 3 | 5.1612 | 675.3815 | 281.2226 | 285.2477 | 2415725.277 | C16H29O2N2 | C12H18O3 | 210.1256 | (±)-Jasmonic Acid | 77026-92-7 | C08491 |
| 4 | 5.68599 | 697.5957 | 273.2175 | 277.2424 | 8681027.886 | C14H29O3N2 | C10H18O4 | 202.1205 | Sebacic acid | 111-20-6 | C08277 |
| 5 | 5.79772 | 703.4345 | 279.1701 | 283.1957 | 1115418.753 | C15H23O3N2 | C11H12O4 | 208.0736 | 3-(3,4-Dimethoxyphenyl)-2-propenoic acid | 14737-89-4 | - |
| 6 | 5.7478 | 700.313 | 201.1965 | 205.2214 | 33134540.24 | C11H25ON2 | C7H14O2 | 130.0994 | Heptanoic acid | - | C17714 |
| 7 | 6.18516 | 727.6614 | 215.2121 | 219.2373 | 6020427.921 | C12H27ON2 | C8H16O2 | 144.115 | Valproic acid | 99-66-1 | C07185 |
| 8 | 7.06233 | 782.5108 | 213.1963 | 217.2216 | 6685916.595 | C12H25ON2 | C8H14O2 | 142.0994 | 2-Octenoic acid | 1871-67-6 | C16653 |
| 9 | 7.26086 | 794.9251 | 299.2331 | 303.2583 | 9534159.567 | C16H31O3N2 | C12H20O4 | 228.1361 | Traumatic acid | 6402-36-4 | C16308 |
| 10 | 7.359 | 801.1323 | 215.2119 | 219.2369 | 126892753.5 | C12H27ON2 | C8H16O2 | 144.115 | Caprylic acid | 124-07-2 | C06423 |
| 11 | 8.24825 | 860.4397 | 259.2385 | 263.2634 | 945941.031 | C14H31O2N2 | C10H20O3 | 188.1412 | 3-Hydroxycapric acid | 14292-26-3 | - |
| 12 | 8.23034 | 859.2451 | 479.3847 | 483.4093 | 658001283.5 | C28H51O4N2 | C24H40O5 | 408.2876 | Ursocholic acid | 2955-27-3 | - |
| 13 | 8.69582 | 890.2892 | 321.2538 | 325.279 | 927330.471 | C19H33O2N2 | C15H22O3 | 250.1569 | Gemfibrozil | 25812-30-0 | C07020 |
| 14 | 8.65198 | 887.3655 | 315.2645 | 319.2897 | 627467.205 | C17H35O3N2 | C13H24O4 | 244.1675 | 1,11-Undecanedicarboxylic acid | 505-52-2 | - |
| 15 | 8.86027 | 901.5199 | 477.3691 | 481.394 | 360059635.9 | C28H49O4N2 | C24H38O5 | 406.2719 | 7-Ketodeoxycholic acid | 911-40-0 | - |
| 16 | 8.845 | 900.2886 | 229.2276 | 233.2525 | 269705482.6 | C13H29ON2 | C9H18O2 | 158.1306 | Nonanoic acid | 112-05-0 | C01601 |
| 17 | 8.93115 | 907.2338 | 463.3898 | 467.4148 | 23192434.53 | C28H51O3N2 | C24H40O4 | 392.2627 | Isoursodeoxycholic acid | 78919-26-3 | - |
| 18 | 9.68509 | 967.8825 | 405.3114 | 409.3365 | 9801175.464 | C24H41O3N2 | C20H30O4 | 334.2144 | PGA2 | 13345-50-1 | C05953 |
| 19 | 10.0853 | 1000.31 | 243.2433 | 247.2683 | 226298335.1 | C14H31ON2 | C10H20O2 | 172.1463 | Capric acid | 334-48-5 | C01571 |
| 20 | 10.0718 | 999.1933 | 255.2434 | 259.2685 | 17200363.13 | C15H31ON2 | C11H20O2 | 184.1463 | Undecylenic acid | 112-38-9 | C12522 |
| 21 | 10.6005 | 1045.931 | 287.2697 | 291.2947 | 1797016.917 | C16H35O2N2 | C12H24O3 | 216.1725 | 3-Hydroxydodecanoic acid | 1883-13-2 | - |
| 22 | 11.2136 | 1100.247 | 257.2591 | 261.2841 | 36445392.9 | C15H33ON2 | C11H22O2 | 186.162 | Undecanoic acid | 112-37-8 | C17715 |
| 23 | 11.1527 | 1094.835 | 357.3117 | 361.3366 | 1297140.783 | C20H41O3N2 | C16H30O4 | 286.2144 | Hexadecanedioic acid | 505-54-4 | C08260 |
| 24 | 12.2634 | 1200.63 | 271.2747 | 275.2995 | 631796857.4 | C16H35ON2 | C12H24O2 | 200.1776 | Lauric acid | 143-07-7 | C02679 |
| 25 | 12.6565 | 1238.7 | 389.3166 | 393.3415 | 10534660.64 | C24H41O2N2 | C20H30O3 | 318.2194 | Leukotriene A4 | 72059-45-1 | C00909 |
| 26 | 13.0005 | 1271.895 | 297.2901 | 301.3152 | 209708440.8 | C18H37ON2 | C14H26O2 | 226.1933 | Myristoleic acid | - | C08322 |
| 27 | 13.2949 | 1300.387 | 285.2903 | 289.3153 | 178623349.7 | C17H37ON2 | C13H26O2 | 214.1933 | Tridecanoic acid | 638-53-9 | C17076 |
| 28 | 13.8206 | 1355.243 | 347.3059 | 351.3309 | 382413033.4 | C22H39ON2 | C18H28O2 | 276.2089 | Stearidonic Acid | 20290-75-9 | C16300 |
| 29 | 14.2578 | 1400.819 | 299.3056 | 303.3303 | 3768894996 | C18H39ON2 | C14H28O2 | 228.2089 | Myristic acid | 544-63-8 | C06424 |
| 30 | 14.7605 | 1451.241 | 349.3215 | 353.3459 | 5404017102 | C22H41ON2 | C18H30O2 | 278.2246 | Alpha-Linolenic acid | 463-40-1 | C06427 |
| 31 | 15.251 | 1500.435 | 313.3218 | 317.3466 | 1951178740 | C19H41ON2 | C15H30O2 | 242.2246 | Pentadecylic acid | 1002-84-2 | C16537 |
| 32 | 15.4501 | 1520.333 | 327.3372 | 331.3622 | 27447912.53 | C20H43ON2 | C16H32O2 | 256.2402 | Isopalmitic acid | 32844-67-0 | - |
| 33 | 16.5989 | 1635.161 | 353.353 | 357.3772 | 23365852943 | C22H45ON2 | C18H34O2 | 282.2559 | Oleic acid | 112-80-1 | C00712 |
| 34 | 17.0331 | 1678.563 | 403.3687 | 407.3935 | 275477409.8 | C26H47ON2 | C22H36O2 | 332.2715 | Adrenic Acid | 28874-58-0 | C16527 |
| 35 | 17.2509 | 1700.337 | 341.3531 | 345.3779 | 784668140.5 | C21H45ON2 | C17H34O2 | 270.2559 | Margaric acid | 506-12-7 | - |
| 36 | 17.2683 | 1702.098 | 379.3685 | 383.3936 | 513217501.7 | C24H47ON2 | C20H36O2 | 308.2715 | Eicosadienoic acid | 2091-39-6 | C16525 |
| 37 | 18.0716 | 1783.407 | 355.3687 | 359.3933 | 5234439295 | C22H47ON2 | C18H36O2 | 284.2715 | Stearic acid | 57-11-4 | C01530 |
| 38 | 18.5381 | 1830.634 | 381.3843 | 385.4089 | 2586073263 | C24H49ON2 | C20H38O2 | 310.2872 | Cis-8-Eicosenoic acid | 5561-99-9 | C16526 |
| 39 | 20.1271 | 2000.403 | 383.4001 | 387.4248 | 1175004095 | C24H51ON2 | C20H40O2 | 312.3028 | Arachidic acid | 506-30-9 | C06425 |
| 40 | 20.3048 | 2020.325 | 409.4156 | 413.4403 | 1297628909 | C26H53ON2 | C22H42O2 | 338.3185 | Erucic acid | 112-86-7 | C08316 |
| 41 | 21.0163 | 2100.104 | 397.4155 | 401.4406 | 329740227.1 | C25H53ON2 | C21H42O2 | 326.3185 | Heneicosanoic acid | 2363-71-5 | - |
| 42 | 21.7877 | 2200.987 | 411.4315 | 415.4561 | 932694976 | C26H55ON2 | C22H44O2 | 340.3341 | Docosanoic acid | 112-85-6 | C08281 |
